# Elucidating the roles of *SOD3* correlated genes and reactive oxygen species in rare human diseases using a bioinformatic-ontology approach

**DOI:** 10.1371/journal.pone.0313139

**Published:** 2024-10-31

**Authors:** Mark Stanworth, Shu-Dong Zhang

**Affiliations:** Personalised Medicine Centre, School of Medicine, Ulster University, C-TRIC Building, Altnagelvin Hospital, Derry, Londonderry, Northern Ireland; Novartis Institutes for BioMedical Research, UNITED STATES OF AMERICA

## Abstract

Superoxide Dismutase 3 (SOD3) scavenges extracellular superoxide giving a hydrogen peroxide metabolite. Both Reactive Oxygen Species diffuse through aquaporins causing oxidative stress and biomolecular damage. *SOD3* is differentially expressed in cancer and this research utilises Gene Expression Omnibus data series GSE2109 with 2,158 cancer samples. Genome-wide expression correlation analysis was conducted with *SOD3* as the seed gene. Categorical *SOD3* Pearson Correlation gene lists incrementing in correlation strength by 0.01 from ρ≥|0.34| to ρ≥|0.41| were extracted from the data. Positively and negatively *SOD3* correlated genes were separated for each list and checked for significance against disease overlapping genes in the ClinVar and Orphanet databases via Enrichr. Disease causal genes were added to the relevant gene list and checked against Gene Ontology, Phenotype Ontology, and Elsevier Pathways via Enrichr before the significant ontologies containing causal and non-overlapping genes were reviewed with a literature search for possible disease and oxidative stress associations. 12 significant individually discriminated disorders were identified: Autosomal Dominant Cutis Laxa (p = 6.05x10^-7^), Renal Tubular Dysgenesis of Genetic Origin (p = 6.05x10^-7^), Lethal Arteriopathy Syndrome due to Fibulin-4 Deficiency (p = 6.54x10^-9^), EMILIN-1-related Connective Tissue Disease (p = 6.54x10^-9^), Holt-Oram Syndrome (p = 7.72x10^-10^), Multisystemic Smooth Muscle Dysfunction Syndrome (p = 9.95x10^-15^), Distal Hereditary Motor Neuropathy type 2 (p = 4.48x10^-7^), Congenital Glaucoma (p = 5.24x210^-9^), Megacystis-Microcolon-Intestinal Hypoperistalsis Syndrome (p = 3.77x10^-16^), Classical-like Ehlers-Danlos Syndrome type 1 (p = 3.77x10^-16^), Retinoblastoma (p = 1.9x10^-8^), and Lynch Syndrome (p = 5.04x10^-9^). 35 novel (21 unique) genes across 12 disorders were identified: *ADNP*, *AOC3*, *CDC42EP2*, *CHTOP*, *CNN1*, *DES*, *FOXF1*, *FXR1*, *HLTF*, *KCNMB1*, *MTF2*, *MYH11*, *PLN*, *PNPLA2*, *REST*, *SGCA*, *SORBS1*, *SYNPO2*, *TAGLN*, *WAPL*, *and ZMYM4*. These genes are proffered as potential biomarkers or therapeutic targets for the corresponding rare diseases discussed.

## Introduction

The basic cellular mechanisms of oxidative stress are fairly well established but the role of Reactive Oxygen Species (ROS) scavengers as well as small molecule ligands in disease is rarely examined. Superoxide dismutases (SODs) are the prevalent ROS scavengers. SOD1 (Cu/Zn) and SOD2 (Mn) are localised to the cytosol and mitochondria respectively whereas SOD3 (Cu/Zn) is localized to the extracellular matrix and blood. This paper is focusses on *SOD3* due to the accessibility of its product as a therapeutic target and the potential for dysfunction to align with the Oxidative Stress Theory of disease. Oxidative stress is an imbalance of reactive oxygen species leading to molecular damage [1]. It was hypothesized to play a role in aging in the 1950’s [2], and has since been suspected as causal in thousands of disorders [3]. The Oxidative Stress Theory of Disease assumes cellular metabolism of oxygen (O_2_) produces reactive oxygen species (ROS) such as superoxide (O_2_^-^) and Hydrogen Peroxide (H_2_O_2_), and biomolecular damage caused by dysregulation of these cytotoxic species can be a causal factor in disease [4].

O_2_^-^ is generated by processes such as cellular respiration and enzymes such as the NADPH oxidases. It is also elevated indirectly by ionizing radiation such as radiotherapy. Ironically, excessive O_2_^-^ can lead to protein and DNA repair mechanism damage, a common feature of cancers. SODs oxidise O_2_^-^ to H_2_O_2_. However, elevated H_2_O_2_ can also cause disorders such as ulcerative colitis, sepsis, angiopathies, systemic lupus erythematosus, and multiple organ failure [5].

Disease states associated with SOD3 include diabetes mellitus, ischemic stroke, and renal disease, and SOD3 has been suggested as a potential target for several inflammatory diseases with mimetics a potential therapeutic for Breast Cancers. Copper chelation therapy may aid SOD3 in removing O_2_^-^ and be of therapeutic benefit to cancer patients [6]. However, there is the caveat that while *SOD3* is generally downregulated in cancers, elevated SOD3 levels have been associated with a poor lung cancer prognosis [7].

SOD3 is a homo-tetramer, with each subunit containing a binding site for Copper (Cu^2+^). Redox reactions between Cu^2+^ and O_2_^-^ lead to the oxidation of O_2_^-^ to O_2_ with Cu^2+^ reduced to Cu^+^ in the process (Eq 1).


$${\text{SOD3-Cu}}^{2+}+{{\text{O}}_{2}}^{-}\to {\text{SOD3-Cu}}^{2+}+{\text{O}}_{2}+{\text{e}}^{-}\to {\text{SOD3-Cu}}^{+}+{\text{O}}_{2}$$ (Eq 1)

An electron is then donated by Cu^+^ to another O_2_^-^ allowing protonation of the latter into H_2_O_2_ (Eq 2).


$${\text{SOD3-Cu}}^{+}+{{\text{O}}_{2}}^{-}+2{\text{H}}^{+}\to {\text{SOD3-Cu}}^{2+}+{\text{e}}^{-}+{{\text{O}}_{2}}^{-}+2{\text{H}}^{+}\to {\text{SOD3-Cu}}^{2+}+{\text{H}}_{2}{\text{O}}_{2}$$ (Eq 2)

Glutathione is oxidised as glutathione peroxidase catalyses H_2_O_2_ into H_2_O.

It is often assumed SOD3 has the singular function to address O_2_^-^ induced oxidative stress, but SOD3 has functional sites within the C-terminal region and can bind with collagen (COL1) and fibulin-5 (FBLN5) of the extracellular matrix. The implications of these interactions are poorly researched, and their potential role in disease aetiology remains undetermined.

Cu^2+^ may also play a role in the management of oxidative stress as a cofactor for both SOD1 and SOD3. Cations held within SOD3 interact with four amino acids: HIS96, HIS98, HIS113, and HIS163. Variants affecting codons for these amino acids could affect the ability of SOD3 to act as an antioxidant and limit copper scavenging activities potentially contributing to copperiedus.

The SODs appear to have evolved across species in a process of convergent evolution leading to variation in SOD location and structure. The *SOD3* gene is located on chromosome 4 in humans but chromosome 5 in mice, and they differ in transcription factor binding site structure [8]. While they share the same function mouse *SOD3* only shares 60% homology with the human gene and product with major differences in the NH2-terminus [9]. This increases the likelihood of requiring expensive transgenic model organisms in studies of its therapeutic potential. SOD and reduced glutathione are also available as an over-the-counter supplement which hinders research funding due to limited financial return.

To support the Oxidative Stress Theory of Disease an association must be made between oxidative stress and disease. We tested the hypothesis that *SOD3* correlated genes and oxidative stress play a significant role in disease and presentation. This research sought to advance understanding of the role of SOD3, O_2_^-^, and H_2_O_2_ in disease using a bioinformatic and ontological approach. Novel genes were sought for their products as potential molecules of interest for future research of biomarkers, and/or therapeutic targets. This was done utilising Pearson Correlation, gene ontology, and a literature review. Potential roles and pathways of *SOD3* correlated genes and oxidative distress in diseases was investigated through a literature review.

## Materials and methods

### Materials

A HP Envy x360 Convertible 15-ee0xxx with an AMD Ryzen 7 4700U processor (8 x 2.0GHz) and 16GB RAM running on a Windows 10 operating system was used for data analysis. The software utilised were ‘7-zip’ (22.00(x64)) to extract CEL files; R (version 4.2.3) with the library containing ‘affy’ (version1.74.0), ‘pvclust’ (version 2.2–0), and ‘jetset’ (version 3.4.0) for Robust Multi-Array normalisation, robustness testing, and identification of the ‘best’ duplicated probe expression respectively; Microsoft Excel (Office 365) for calculating Jarque-Bera normality test statistic, χ^2^ test, Pearson correlation, and two tail t-test calculations. Internet connection and browser were required to access web content.

### Methods

Co-expressed genes are often functionally related [10]. From an input list of correlated genes, gene set enrichment analysis (GSEA) identifies gene-sets that are overrepresented under certain conditions and aid biological interpretation [11]. We used expression data to identify gene expressions correlated with *SOD3*, then online GSEA tools were used to identify disorders and ontologies associated with the co-expressed genes. While these methods highlight statistical significance, a literature search to identify potential pathways and interactions was performed to derive conclusions by inductive reasoning.

The standard significance threshold of α = 0.05 was adopted. Formulae and coding used in this research is reproduced in S1 File. The methods including formulae are also available at doi.org/10.17504/protocols.io.rm7vzxp14gx1/v1.

#### Expression preparation

*SOD3* is downregulated in cancer cells and the differential expression enhances the likelihood of correctly identifying correlated and functionally related genes. Gene Expression Omnibus expression profiling datasets were interrogated for “cancer” and filtered by “Homo sapiens” and “Expression profiling by array”. As *SOD3* is differentially expressed in cancers, series GSE2109 [12] was selected for the sample size (n = 2,158) and probe count (n = 54,676) giving 117,990,808 expression values which approached the viable upper limit for hardware and software performance. The RAW file was downloaded and CEL files extracted using ‘7-zip’ software due to a RAM limit issue arising when utilising the ‘GEOquery’ package in R.

Robust Multiarray Averaging (RMA) normalisation is considered a reliable method for Affymetrix microarray data despite the possibility of artefacts [13]. The statistical analysis required data to approximate a normal distribution, and RMA normalisation was performed using the R ‘Affy’ package.The ‘pvclust’ package was used to test for robustness. Probe expressions were exported as a tab delimited file to minimise file size, ready for import into Excel.

#### *SOD3* expression correlations

Correlations between *SOD3* expression and all other gene expressions were a primary measure of interest. Pearson correlations are directional thus discriminate positively and negatively correlated gene expressions. Probe expressions from the tab delimited file were imported into Microsoft Excel where the Pearson correlation (ρ) between the reference *SOD3* probe (205236_x_at) and all other probes were calculated. A two-tail t-test was then applied to the correlation to determine statistical significance. After RMA normalisation was performed, the Jarque-Bera normality test with corresponding χ^2^ test with two degrees of freedom were also calculated to verify probe expressions conformed to a normal distribution.

#### Data cleaning

The annotation table for GPL570 (https://www.ncbi.nlm.nih.gov/geo/query/acc.cgi?acc=GPL570) identified a single *SOD3* probe. For duplicated gene probes the R package ‘jetset’ was used to identify the optimal probe using hgu133plus2 chip data and suboptimal duplicates excluded. Probes failing the Jarque-Bera normality test / χ^2^ test for significance at α = 0.05 were excluded (Table 1). The alpha value was Bonferroni corrected giving the adjusted value α_cor_ = 2.3x10^-5^ for Pearson correlation significance, expressions with a p-value greater than this were excluded.

**Table 1:** Probe / gene exclusion flow.

Probe IDs / Genes	Exclusion criteria	Excluded
n = 54,675	Failed Jarque Bera normality test / significance	n = 343
n = 54,332	Failed Pearson Correlation two-tail t-test (α_cor_ = 2.3x10^-5^)	n = 21,693
n = 32,639	Failed Pearson rho threshold (ρ < 0.34)	n = 32,504
n = 135	No Gene Symbol for Probe ID	n = 1
n = 134	Non-specific Probe IDs	n = 9
n = 125	Non-coding Genes	n = 7
n = 118	Duplicated Genes	n = 18
n = 100 (List 8)	ρ < 0.34	n = 32
n = 68 (List 7)	ρ < 0.35	n = 26
n = 42 (List 6)	ρ < 0.36	n = 8
n = 34 (List 5)	ρ < 0.37	n = 14
n = 20 (List 4)	ρ < 0.38	n = 7
n = 13 (List 3)	ρ < 0.39	n = 4
n = 9 (List 2)	ρ < 0.40	n = 4
n = 5 (List 1)	ρ < 0.41	

Exclusion data from the criteria outlined in text.

Preliminary testing of Pearson correlation thresholds to create SOD3-correlated gene lists for entry into ClinVar and Orphanet via Enrichr (https://maayanlab.cloud/Enrichr/) indicated absolute Pearson correlations between ρ = |0.34| and ρ = |0.41| would be optimal for gene list associated disorder discovery. Correlations of ρ≤|0.34| were excluded.

Gene symbols for the remaining probes were identified from GPL570 annotation table and manually updated using the HGNC database (https://www.genenames.org/). Probes with no gene ID, nonspecific / promiscuous probes, probes for non-coding genes, and sub-optimal duplicated genes were excluded. This resulted list of 100 *SOD3* correlated genes at ρ≥|0.34| for further investigation.

To verify reliability the gene list expressions were tested for robustness using the ‘pvclust’ package in R. Correlation distance was used with bootstrapping (nboot = 1,000) and robustness calculated at 95% confidence.

#### Gene lists

By incrementally increasing the correlation threshold by 0.01 from ρ≥|0.34| to ρ≥|0.41| the gene list was categorically divided into 8 SOD3-correlated gene lists which were numbered in ascending order by gene count (Table 1). Full lists were then sub-divided into signed lists of positive and negative correlation denoted X^+^ and X^-^ giving 24 gene lists.

#### Significant disorders

All 8 SOD3-correlated gene lists and their sub-divided signed lists were individually entered into Enrichr to identify disorders with significant gene overlap in ClinVar 2019 and Orphanet Augmented 2021 databases. Disorders without statistically significant overlap were excluded and to maintain *SOD3* relevance, only disorders arising from the smallest viable list were considered further. A SOD3-correlated gene list is viable if it returns at least one disorder that meets the inclusion criteria below. First, the overlap between the correlate gene list and a disorder must be statistically significant as determined by an adjusted p value (< 0.05) from the Enrichr analysis. Second, to be included, a disorder needs to have a minimum number of genes in its overlap with the correlate gene list. For a positively correlated list, disorders without *SOD3* plus two other correlate list genes in the overlap were excluded. A negatively correlated list cannot contain *SOD3* in it by definition but required a disorder to overlap with at least three SOD3-correlated genes for the disorder to be included.

Orphanet (https://www.orpha.net/) was cross referenced with the Online Mendelian Inheritance in Man® (https://www.omim.org/) to identify the accepted causal genes for each qualifying disorder. Any disorders which could not be uniquely discriminated by overlap and causal gene(s) were excluded from further consideration.

The qualifying disorders identified for the smallest viable lists were checked for significance in the larger parent lists and the list with the optimal overlap / significance was attached to the disorder.

#### Significant ontologies

For each disorder the correlate gene list with the greatest overlap (with the disorder) and significance had the causal gene(s) added to it and passed into Enrichr to identify Gene Ontology Biological Process and Molecular Function, Human Phenotype Ontology, and Elsevier Pathways. For each disorder significant ontologies containing a causal gene plus a correlate list gene non-overlapping with the disorder were used to identify potential mechanisms and novel genes not previously reported in the literature.

Disorders that could not be discriminated from other disorders by gene overlap or known causal gene(s) were excluded.

#### Literature associations

A literature search for the ontology identified non-overlapping genes and evidence of their interaction with the disorder, causal gene, O_2_^-^, and H_2_O_2_ was undertaken. This served as the basis for a discussion on the potential role of the suspected novel genes, the seed gene *SOD3*, and the metabolites O_2_^-^ and H_2_O_2_. SOD3-correlated list genes non-overlapping with the disorder were considered novel if they were linked to a causal gene by ontology and the literature provided evidence of a possible interaction or mechanism / pathway between the causal gene(s) and the identified non-overlapping gene.

## Results

**Fig 1:**
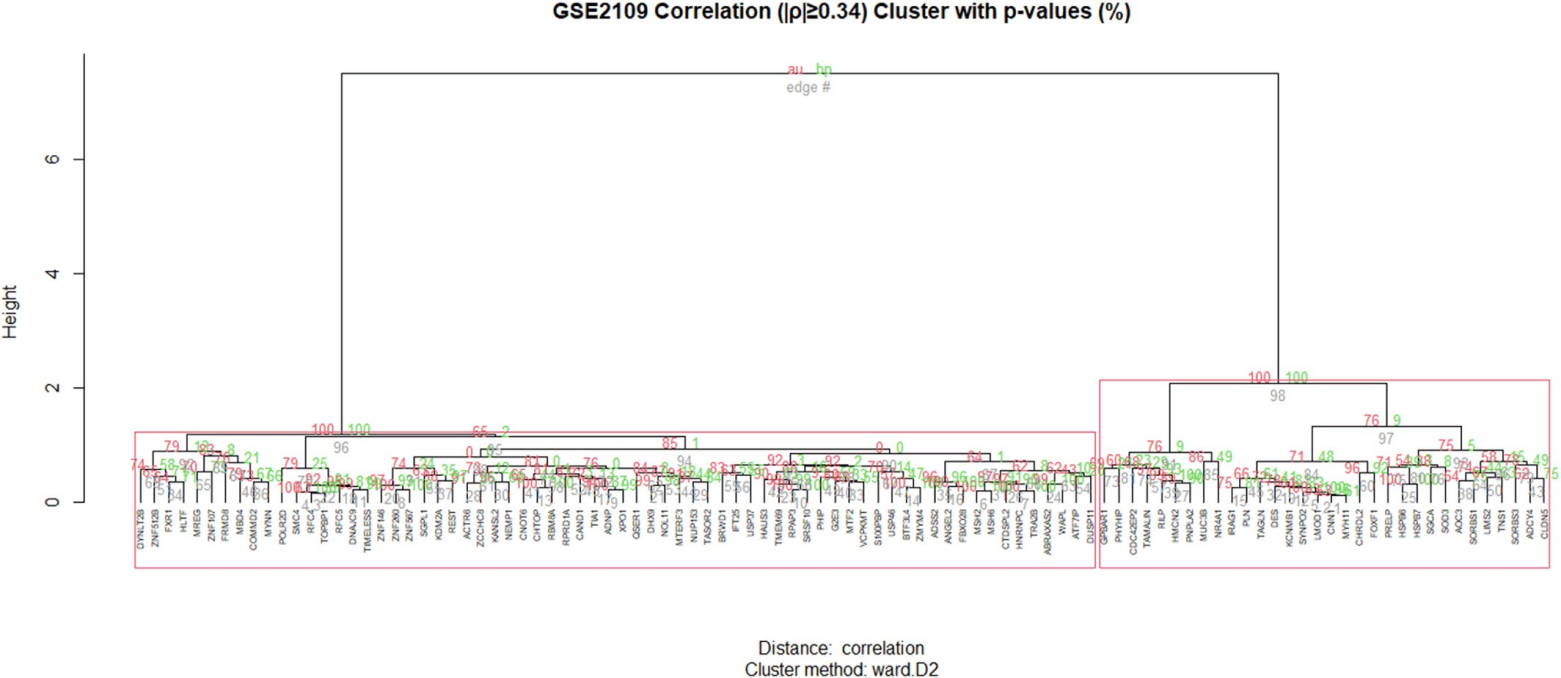
Gene list 8 robustness. The full gene list robustness at 95% confidence using ‘pvclust’ (nboot = 1 000). By incrementally increasing the correlation threshold by 0.01 from ρ≥|0.34| to ρ≥|0.41| the gene list was categorically divided into 8 gene lists which were numbered in ascending order by gene count (Table 1). Full lists were then sub-divided into signed lists of positive and negative correlation denoted X^+^ and X^-^ resulting in 24 gene lists.

After exclusions (Table 1) a total of 100 genes were selected for consideration (ρ≥|0.34|), 32 genes were positively correlated to *SOD3* and 68 negatively correlated. 8 genes had duplicate probes and the ‘best’ probe was identified using the R ‘Jetset’ package; *CAND1* (208838_at), *FBXO28* (202271_at), *HSPB6* (226304_at), *MREG* (219648_at), *MTF2* (209705_at), *MYH11* (201497_x_at), *PLN* (228202_at), *QSER1* (219705_at). Positive and negative correlated genes were exclusively clustered during robustness testing (Fig 1).

### Gene lists

Enrichment analysis utilising ClinVar and Orphanet within Enrichr gave the same overlap and greater statistical significance with fewer genes for the 16 signed lists over the 8 unsigned lists. The unsigned gene lists were thus no longer considered. Gene lists and links to the gene lists on Enrichr are available in S1-S8 Tables in S1 File.

#### Significant disorders

ClinVar returned no disorders meeting criteria for either unsigned or signed lists, however, Orphanet returned 27 disorders which met criteria. The smallest viable positive list was list 1^+^. This list, consisting of 5 genes including SOD3 itself, is enriched and overlapped with 23 disorders; however, 12 disorders could not be uniquely discriminated by overlap and/or causal gene. No eligible disorders could be uniquely discriminated for lists 1-, 2-, or 3-; and only 4 disorders met criteria in list 4^-^ (n = 7). 15 disorders met criteria for further consideration (Table 2).

**Table 2:** Disorders significantly overlapping with SOD3-correlated gene lists.

Disorder	List (ρ/genes)	Overlap	p-value
Lethal Arteriopathy Syndrome due to Fibulin-4 Deficiency	1^+^ (0.41/5)	4	5.52x10^-8^
	**2**^**+**^ **(0.40/9)**	**5**	**6.54x10** ^**-9**^
EMILIN-1-related Connective Tissue Disease	1^+^ (0.41/5)	4	5.52x10^-8^
	**2**^**+**^ **(0.40/9)**	**5**	**6.54x10** ^**-9**^
Megacystis-Microcolon-Intestinal Hypoperistalsis Syndrome	1^+^ (0.41/5)	4	5.52x10^-8^
	**8**^**+**^ **(0.34/32)**	**11**	**3.77x10** ^**-16**^
Multisystemic Smooth Muscle Dysfunction Syndrome	1^+^ (0.41/5)	4	5.52x10^-8^
	**5**^**+**^ **(0.37/19)**	**9**	**9.95x10** ^**-15**^
Congenital Glaucoma	1^+^ (0.41/5)	4	5.70x10^-8^
	**7**^**+**^ **(0.35/29)**	**7**	**5.24x10** ^**-9**^
Holt-Oram Syndrome	1^+^ (0.41/5)	3	1.03x10^-5^
	**4**^**+**^ **(0.38/13)**	**6**	**7.72x10** ^**-10**^
Pancreatic Insufficiency-Anemia-Hyperostosis Syndrome	1^+^ (0.41/5)	3	1.03x10^-5^
	**8**^**+**^ **(0.34/32)**	**9**	**2.42x10** ^**-12**^
Classical-like Ehlers-Danlos Syndrome type 1	1^+^ (0.41/5)	3	1.03x10^-5^
	**8**^**+**^ **(0.34/32)**	**11**	**3.77x10** ^**-16**^
Autosomal Dominant Cutis Laxa	1^+^ (0.41/5)	3	1.03x10^-5^
	**2**^**+**^ **(0.40/9)**	**4**	**6.05x10** ^**-7**^
Renal Tubular Dysgenesis of Genetic Origin	1^+^ (0.41/5)	3	1.03x10^-5^
	**2**^**+**^ **(0.40/9)**	**4**	**6.05x10** ^**-7**^
Distal Hereditary Motor Neuropathy type 2	1^+^ (0.41/5)	3	1.03x10^-5^
	**5**^**+**^ **(0.37/19)**	**5**	**4.48x10** ^**-7**^
Borjeson-Forssman-Lehmann Syndrome	4^-^ (-0.38/7)	3	2.45x10^-4^
	**7**^**-**^ **(-0.35/39)**	**8**	**6.51x10** ^**-7**^
Non-hereditary Retinoblastoma	4^-^ (-0.38/7)	3	2.45x10^-4^
	**6**^**-**^ **(-0.36/21)**	**6**	**1.9x10** ^**-8**^
Hereditary Retinoblastoma	4^-^ (-0.38/7)	3	2.45x10^-4^
	**6**^**-**^ **(-0.36/21)**	**6**	**1.90x10** ^**-8**^
Lynch Syndrome	4^-^ (-0.38/7)	3	2.45x10^-4^
	**7**^**-**^ **(-0.35/39)**	**8**	**5.04x10** ^**-9**^

Values for the smallest viable gene list and the optimal overlapping / most significant gene list (bold) were noted. The optimal list was used for the investigation of the relevant disorder. Hereditary and non-hereditary retinoblastoma have the same causal gene and are considered the same disorder in this paper due to the single OMIM (180200) identifier. No new information for Pancreatic Insufficiency-Anemia-Hyperostosis Syndrome and Borjeson-Forssman-Lehmann Syndrome was discovered.

List 1^+^ had a 4 gene overlap with Lethal Arteriopathy Syndrome due to Fibulin-4 Deficiency (ORPHA:314718, p = 5.52x10^-8^), EMILIN-1-related Connective Tissue Disease (ORPHA:485418, p = 5.52x10^-8^), Megacystis-Microcolon-Intestinal Hypoperistalsis Syndrome (ORPHA:2241, p = 5.52x10^-8^), Multisystemic Smooth Muscle Dysfunction Syndrome (ORPHA:404463, p = 5.52x10^-8^), and Congenital Glaucoma (ORPHA:98976, p = 5.70x10^-8^), and a 3 gene overlap with Holt-Oram Syndrome (ORPHA:392, p = 1.03x10^-5^), Pancreatic Insufficiency-Anemia-Hyperostosis Syndrome ORPHA:199337, p = 1.03x10^-5^), Classical-like Ehlers-Danlos Syndrome type 1 (ORPHA:230839, p = 1.03x10^-5^), Autosomal Dominant Cutis Laxa (ORPHA:90348, p = 1.03x10^-5^), Renal Tubular Dysgenesis of Genetic Origin (ORPHA:97369, 1.03x10^-5^), and Distal Hereditary Motor Neuropathy type 2 (ORPHA:139525, 1.03x10^-5^).

List 4^-^ identified four significant disorders with a 3 gene overlap; Borjeson-Forssman-Lehmann Syndrome (ORPHA:127, p = 2.45x10^-4^), Non-hereditary Retinoblastoma (ORPHA:357034, p = 2.45x10^-4^), Hereditary Retinoblastoma (ORPHA:357027, p = 2.45x10^-4^), and Lynch Syndrome (p = 2.45x10^-4^). These disorders had a greater overlap and significance in larger negative correlation gene lists (Table 2). The two Retinoblastoma entries could not be discriminated as they are otherwise considered identical with the same causal gene but are presented separately here due to the separate Orphanet identifiers.

After testing all lists the parent lists with the optimal significant overlap were linked to the disorders identified by the smallest viable list.

#### Significant ontologies

Causal genes were added to optimal disorder associated lists. Non-overlapping list genes were considered implicated in a disorder if they appeared in the ontologies along with a causal or overlap gene. 31genes (14 unique) were identified as being potentially novel for the 10 disorders in the positive correlation lists, 1 disorder failed to yield results. 9 genes (8 unique) were identified as potentially novel for the 2 disorders in the negative correlation lists with 2 disorders combined and 1 failing to yield results.

**Fig 2:**
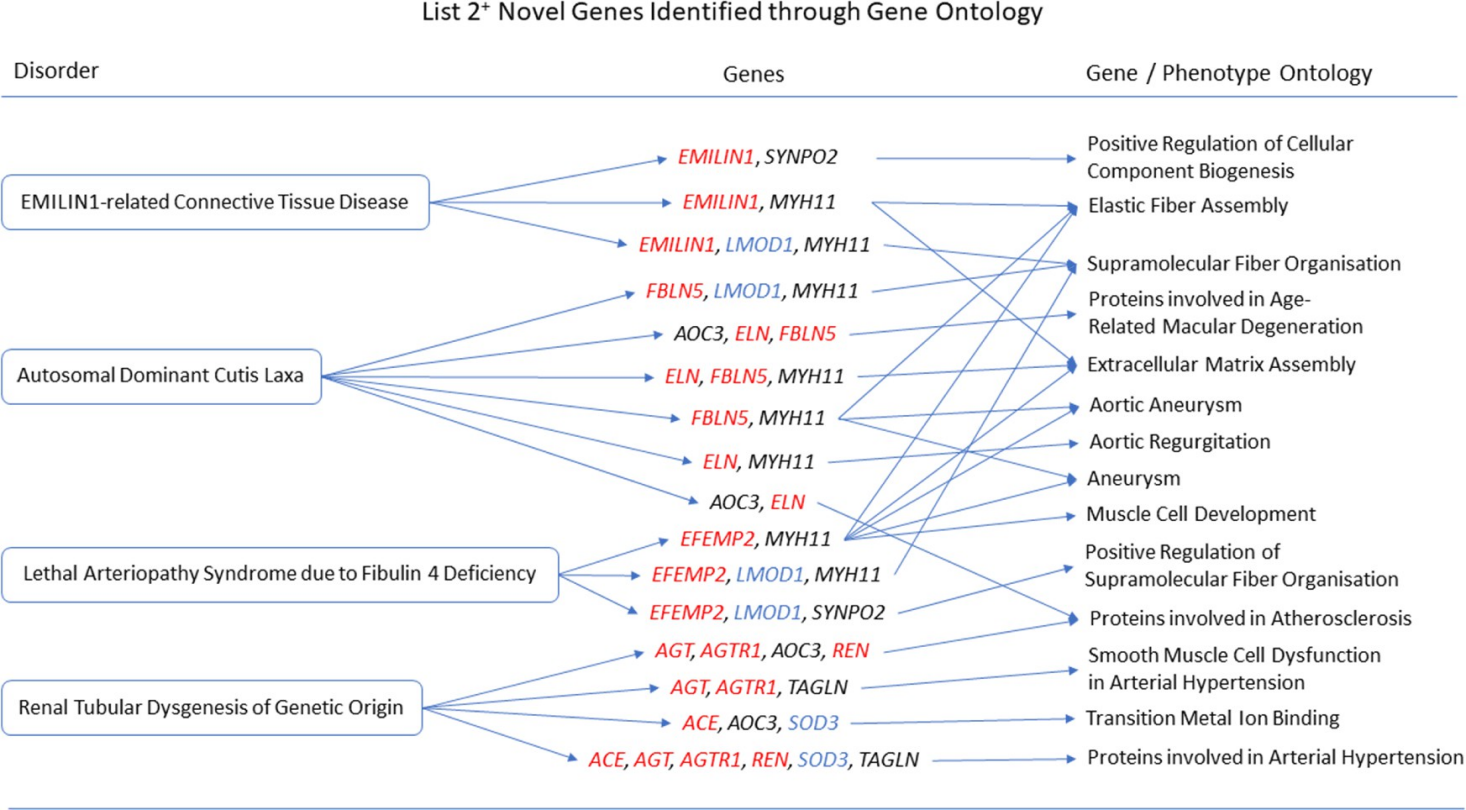
List 2^+^ ontology. Gene Ontologies resulting from list 2^+^ genes in combination with disorder causal genes linked several new, SOD3 correlated genes, to the disorders. Gene colours: Red = causal gene, blue = overlapping gene, black = list gene not causal or overlapping.

**Fig 3:**
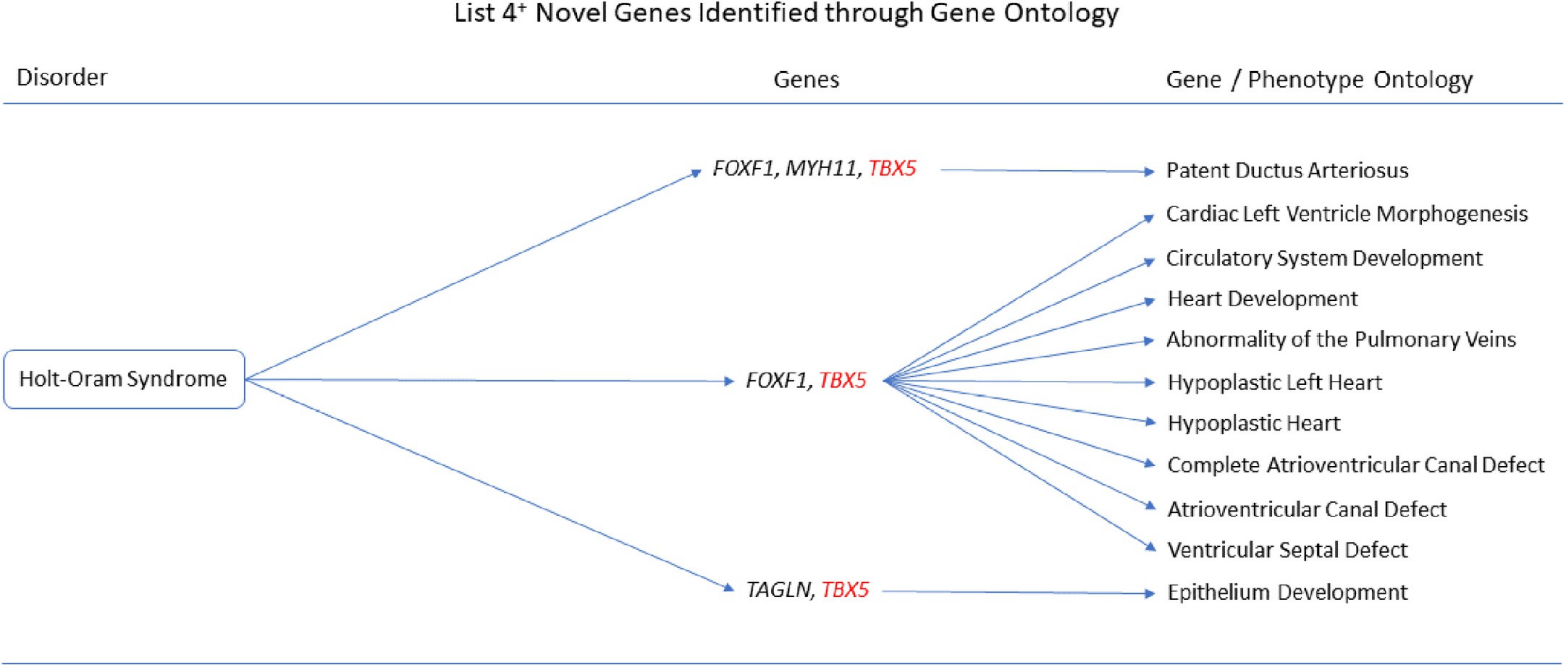
List 4^+^ ontology. Gene Ontologies resulting from list 4^+^ genes in combination with disorder causal genes linked several new, SOD3 correlated genes, to the disorders. Red = causal gene, black = list gene not causal or overlapping. Gene colours: Red = causal gene, black = list gene not causal or overlapping.

**Fig 4:**
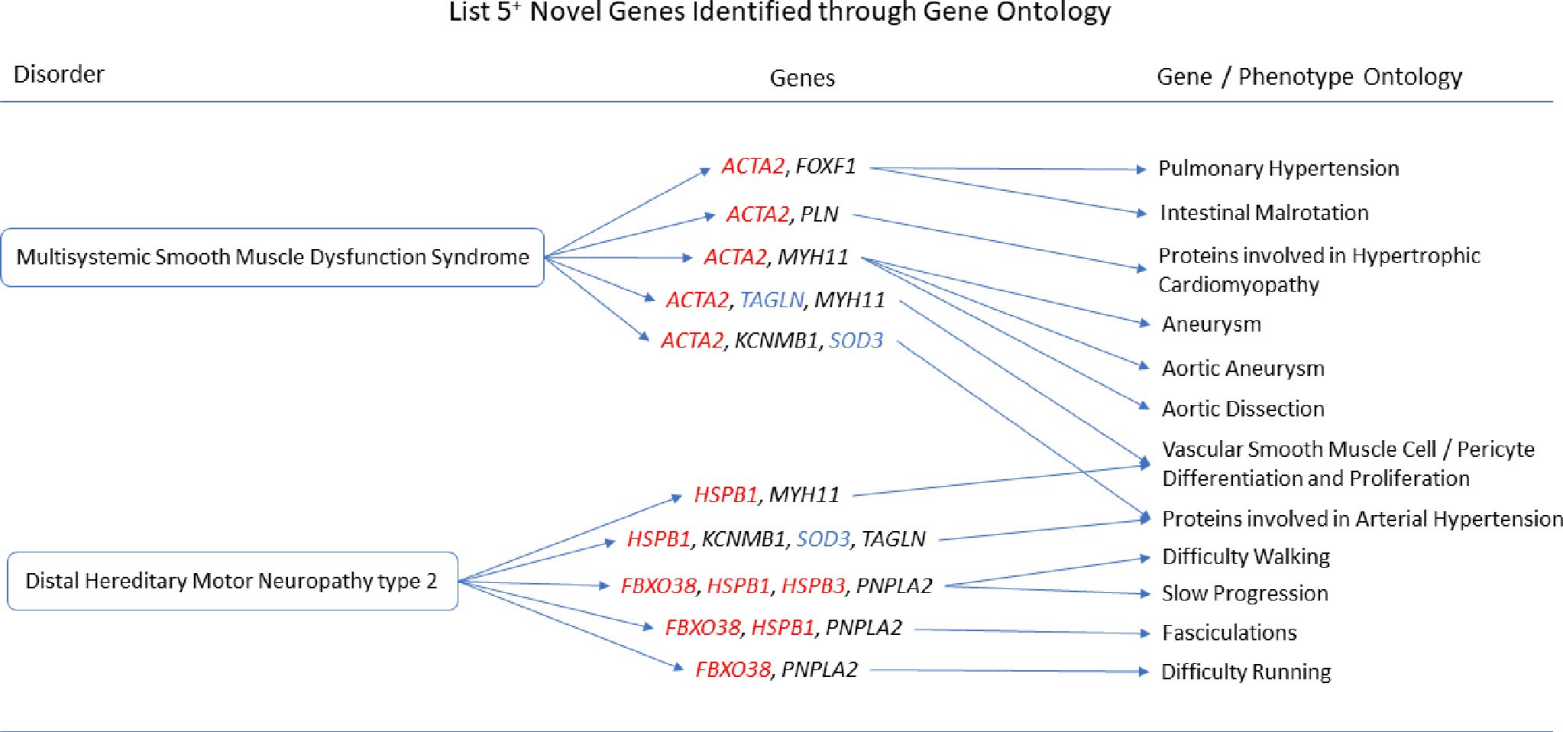
List 5^+^ ontology. Gene Ontologies resulting from list 5^+^ genes in combination with disorder causal genes linked several new, SOD3 correlated genes, to the disorders. Gene colours: Red = causal gene, blue = overlapping gene, black = list gene not causal or overlapping.

**Fig 5:**
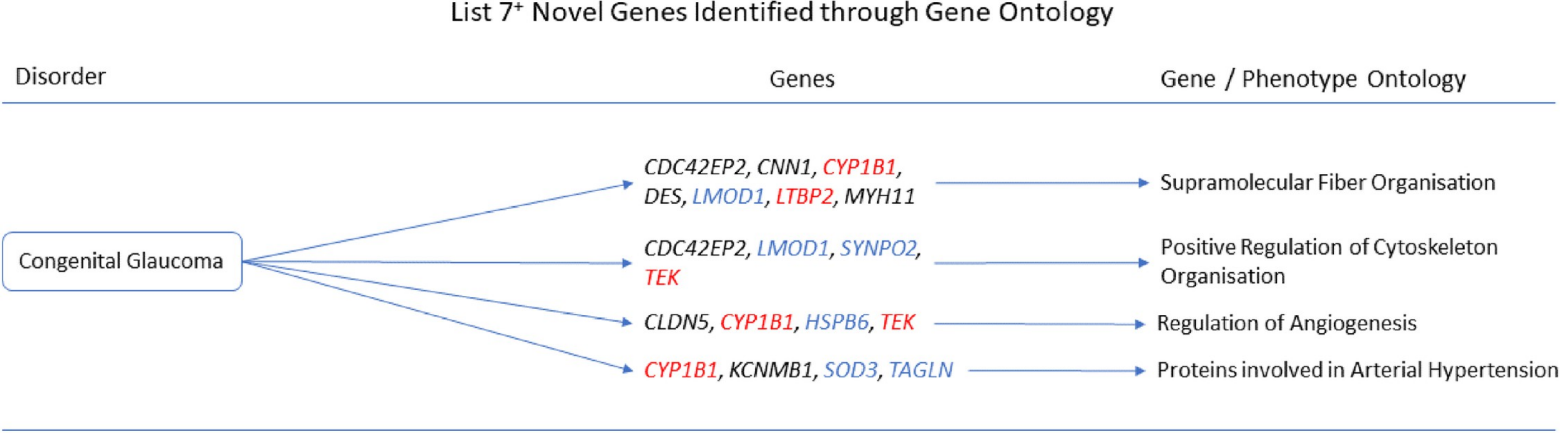
List 7^+^ ontology. Gene Ontologies resulting from list 7^+^ genes in combination with disorder causal genes linked several new, SOD3 correlated genes, to the disorders. Gene colours: Red = causal gene, blue = overlapping gene, black = list gene not causal or overlapping.

**Fig 6:**
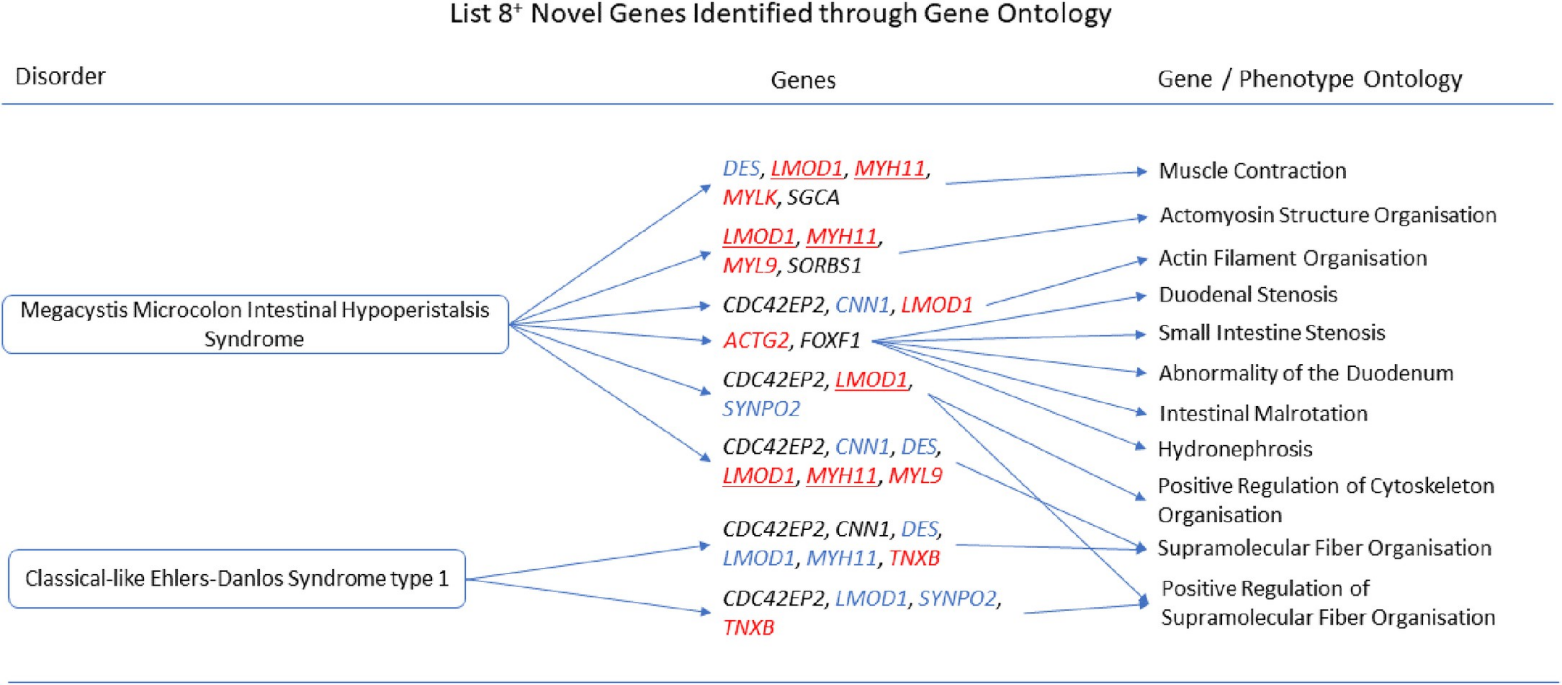
List 8+ ontology. Gene Ontologies resulting from list 8^+^ genes in combination with disorder causal genes linked several new, SOD3 correlated genes, to the disorders. Gene colours: Red = causal gene, red underlined = causal and overlapping gene, blue = overlapping gene, black = list gene not causal or overlapping.

**Fig 7:**
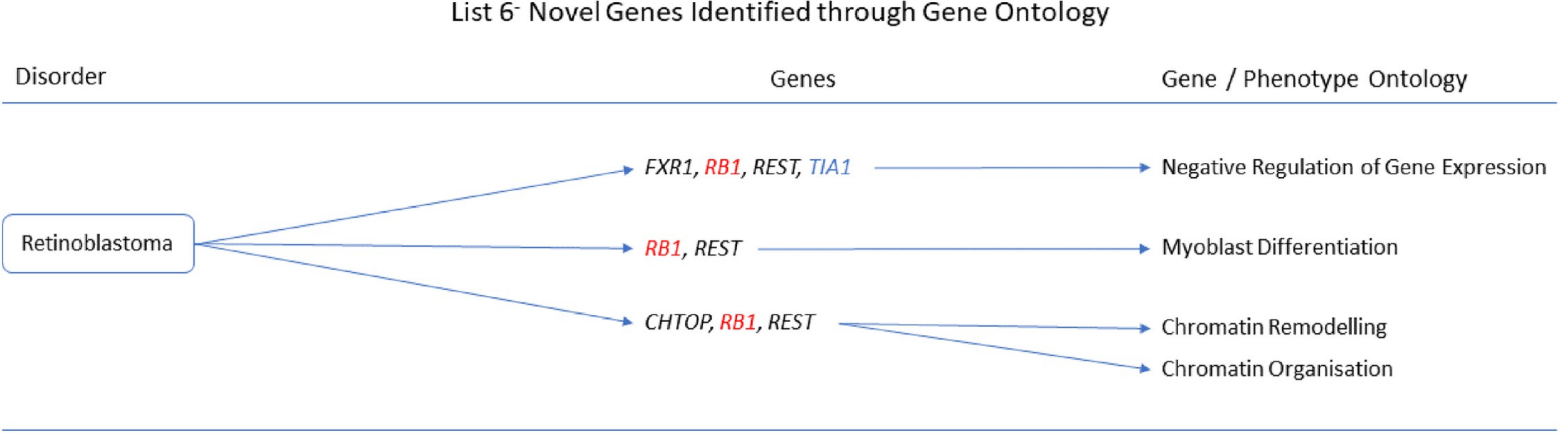
List 6^-^ ontology. Gene Ontologies resulting from list 6^-^ genes in combination with disorder causal genes linked several new, SOD3 correlated genes, to the disorders. Gene colours: Red = causal gene, blue = overlapping gene, black = list gene not causal or overlapping.

**Fig 8:**
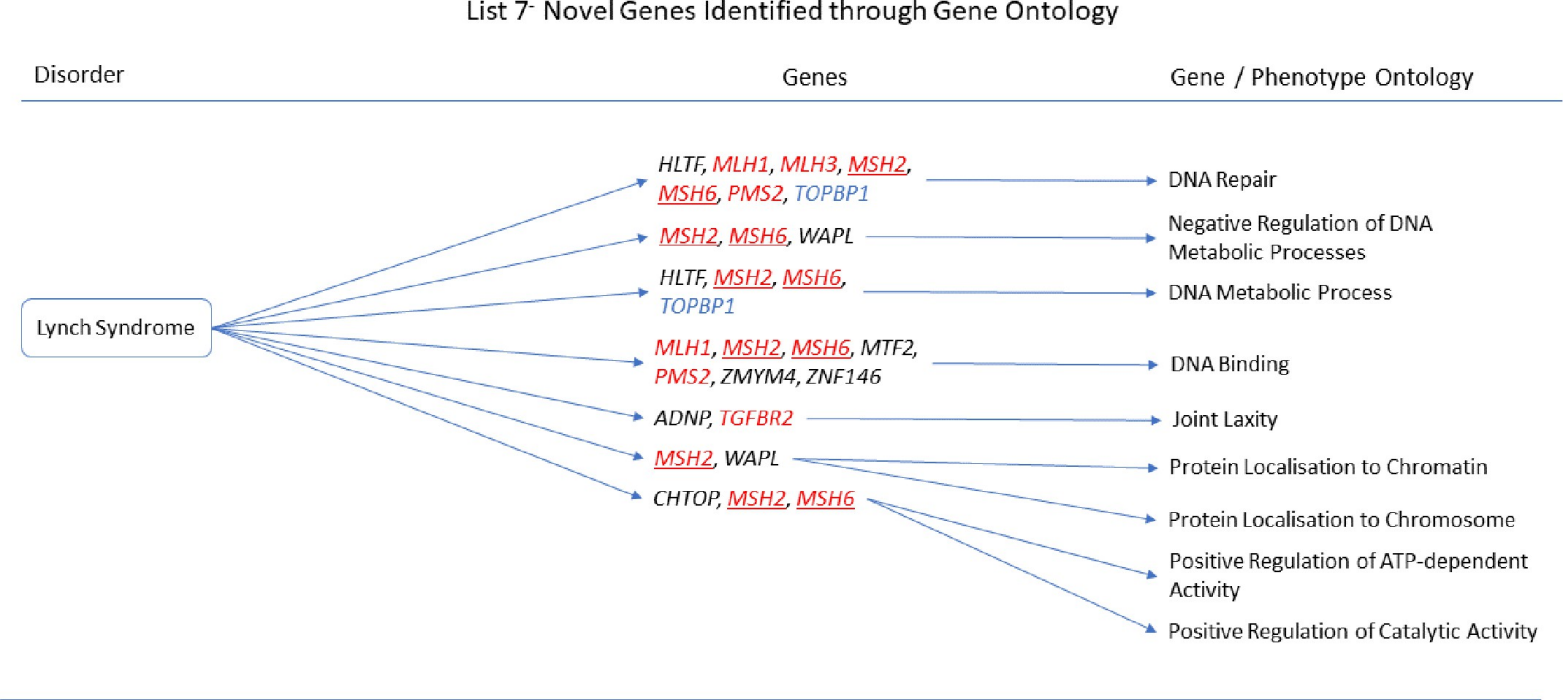
List 7^-^ ontology. Gene Ontologies resulting from list 7^-^ genes in combination with disorder causal genes linked several new, SOD3 correlated genes, to the disorders. Gene colours: Red = causal gene, red and underlined = causal and overlapping gene, blue = overlapping gene, black = list gene not causal or overlapping.

The gene ontologies for list 2^+^ linked several genes to disorders. *MYH11* and *SYNPO2* linked to EMILIN1-related Connective Tissue Disease, *AOC3* and *MYH11* linked to Autosomal Dominant Cutis Laxa, *MYH11* and *SYNPO2* linked to Lethal Arteriopathy Syndrome due to Fibulin-4 Deficiency, while *AOC3* and *TAGLN* linked to Renal Tubular Dysgenesis of Genetic Origin (Fig 2). The gene ontologies for list 4^+^ linked *FOXF1*, *MYH11*, and *TAGLN* to Holt-Oram Syndrome (Fig 3). The gene ontologies for list 5^+^ linked *FOXF1*, *KCNMB1*, *MYH11*, and *PLN* to Multisystemic Smooth Muscle Dysfunction Syndrome. *KCNMB1*, *MYH11*, *PNPLA2*, and *TAGLN* linked to Distal Hereditary Motor Neuropathy type 2 (Fig 4). The gene ontologies for list 7^+^ linked the non-overlapping genes *CDC42EP2*, *CLDN5*, *CNN1*, *DES*, *KCNMB1*, and *MYH11* to Congenital Glaucoma (Fig 5). The gene ontologies for list 8^+^ linked *CDC42EP2*, *FOXF1*, *SGCA*, and *SORBS1* to Megacystis-Microcolon-Intestinal Hypoperistalsis Syndrome while *CDC42EP2* and *CNN1* linked to Classical-like Ehlers-Danlos Syndrome (Fig 6). Hereditary and Non-hereditary Retinoblastoma are listed as a single disorder in OMIM (Retinoblastoma, OMIM:180200) and was considered a single disorder here also. The gene ontologies for list 6^-^ linked *CHTOP*, *FXR1*, and *REST* to Retinoblastoma (Fig 7). The gene ontologies for list 7^-^ linked *CHTOP*, *ADNP*, *HLTF*, *WAPL*, *ZMYM4*, and *ZNF146* to Lynch Syndrome (Fig 8).

#### Autosomal Dominant Cutis Laxa

Adding causal genes *ELN*, *FBLN5*, and *ALDH18A1* to list 2^+^ (n = 9+3) revealed *MYH11* with at least one causal gene to be associated with Extracellular Matrix Assembly (GO:0085029, q = 6.477x10^-6^), Elastic Fiber Assembly (GO:0048251, q = 1.434x10^-4^), Supramolecular Fiber Organisation (GO:0097435, q = 1.166x10^-3^), and Vascular Smooth Muscle Cell/Pericyte Differentiation and Proliferation. *AOC3* was associated with Proteins Involved in Age-Related Macular Degeneration (q = 9.982x10^-4^) and Proteins involved in Atherosclerosis (q = 1.975x10^-2^).

#### Lethal Arteriopathy Syndrome due to Fibulin 4 Deficiency

Adding the causal gene, *EFEMP2* (previously Fibulin-4), to list 2^+^ (n = 9+1) revealed *MYH11* with *EFEMP2* to be associated with Elastic Fiber Assembly (GO0048251, q = 1.854x10^-4^), Extracellular Matrix Assembly (GO:0085029, q = 5.588x10^-4^), Muscle Cell Development (GO:0055001, q = 1.522x10^-3^), and Supramolecular Fiber Organisation (GO:0097435, q = 4.749x10^-3^) as well as being associated with the Aortic Aneurysm Phenotype (HP:0004942, q = 4.815x10^-4^). *SYNPO2* with *EFEMP2* was associated with Positive Regulation of Supramolecular Fiber Organisation (GO:1902905, q = 3.451x10^-4^).

#### EMILIN1-related Connective Tissue Disease

Adding the causal gene, *EMILIN1*, to list 2^+^ (n = 9+1) revealed *MYH11* with *EMILIN1* to be associated with Elastic Fiber Assembly (GO:0048251, q = 3.3x10^-4^), Extracellular Matrix Assembly (GO:0085029, q = 1.478x10^-3^), supramolecular Fiber Organisation (GO:0097435, q = 1.213x10^-2^), and Vascular Smooth Muscle Cell/Pericyte Differentiation and Proliferation (q = 8.084x10^-4^). *SYNPO2* with *EMILIN1* was associated with Positive Regulation of Cellular Component Biogenesis (GO:0044089, q = 1.213x10^-2^).

#### Renal Tubular Dysgenesis of Genetic Origin

Adding the causal genes *ACE*, *AGT*, *AGTR1*, and *REN* to list 2^+^ (n = 9+4) revealed *AOC3* with at least one causal gene to be associated with Transition Metal Ion Binding (GO:0046914, q = 2.288x10^-2^), and Atherosclerosis (q = 2.821x10^-5^). *TAGLN* with at least one causal gene was associated with Arterial Hypertension (q = 7.739x10^-8^) and Smooth Muscle Cell Dysfunction in Arterial Hypertension (q = 2.203x10^-5^).

#### Holt-Oram Syndrome

Adding the causal gene, *TBX5*, to list 4^+^ (n = 13+1) revealed *FOXF1* with *TBX5* to be associated with Cardiac Left Ventricle Morphogenesis (GO:0003214, q = 4.346x10^-3^), Circulatory System Development (GO:0072359, q = 2.665x10^-2^), Heart Development (GO:0007507, q = 2.665x10^-2^), Abnormality of the Pulmonary Veins (HP:0004283, q = 9.239x10^-4^), Hypoplastic Heart (HP:0001961, q = 9.239x10^-4^), Complete Atrioventricular Canal Defect (HP:0001674, q = 9.239x10^-4^), Atrioventricular Canal Defect (HP:0006695, q = 1.162x10^-3^), and Ventricular Septal Defect (HP:0001629, q = 2.388x10^-2^). *FOXF1* and *MYH11* with *TBX5* were implicated in Patent Ductus Arteriosus (HP:0001643, q = 9.239x10^-4^). *TAGLN* with *TBX5* was associated with Epithelium Development (GO:0060429, q = 2.665x10^-2^).

#### Multisystemic Smooth Muscle Dysfunction Syndrome

Adding the causal gene, *ACTA2*, to list 5^+^ (n = 19+1) revealed *MYH11* with *ACTA2* to be associated with Aortic Aneurysm (HP:0004942, q = 2.313x10^-3^), Aortic Dissection (HP:0002647, q = 5.629x10^-3^), and Aneurysm (HP:0002647, q = 8.754x10^-3^). *FOXF1* with *ACTA2* was associated with Intestinal Malrotation (HP:0002566, q = 1.351x10^-2^) and Pulmonary Hypertension (HP:0002092, q = 2.318x10^-2^). *PLN* with *ACTA2* was associated with Hypertrophic Cardiopathy (q = 1.624x10^-2^). *KCNMB1* with *ACTA2* was associated with Arterial Hypertension (q = 1.624x10^-2^). *TAGLN* with *ACTA2* was hypothetically associated with TRPM4/6/7/8 Signalling in Arterial Hypertension (Hypothesis) (q = 1.016x10^-2^), Smooth Muscle Cell Dysfunction in Arterial Hypertension (q = 1.334x10^-2^), and TGF-Beta Family in Epithelial Mesenchymal Transition in Cancer (q = 1.878x10^-2^). *MYH11* and *TAGLN* with *ACTA2* were implicated in Vascular Smooth Muscle Cell/Pericyte Differentiation and Proliferation (5.689x10^-5^). *CNN1* and *TAGLN* with *ACTA2* were implicated in Smooth Muscle Cell Dysfunction in Pulmonary Hypertension (q = 2.622x10^-3^).

#### Distal Hereditary Motor Neuropathy type 2

Adding the causal genes, *HSPB1*, *HSPB3*, *HSPB8*, and *FBXO38*, to list 5^+^ (n = 19+4) revealed *PNPLA2* with at least one causal gene to be associated with Difficulty Walking (HP:0002335, q = 2.830x10^-6^), Slow Progression (HP:0003677, q = 7.000x10^-5^), Fasciculations (HP:0002380, q = 1.043x10^-4^), and Difficulty Running (HP:0009046, q = 6.651x10^-4^).

#### Congenital Glaucoma

When the causal genes *CYP1B1*, *LTBP2*, and *TEK* were added to list 7^+^ (n = 29+3) *CDC42EP2* with at least one causal gene was associated with Positive Regulation of Cytoskeleton Organisation (GO:0051495, q = 1.371x10^-3^). *CLDN5* was implicated in Regulation of Angiogenesis (GO:0045765, q = 1.416x10^-2^). *MYH11* was implicated in Vascular Smooth Muscle Cell/Pericyte Differentiation and proliferation (q = 2.083x10^-2^). *KCNMB1* was implicated in Arterial Hypertension (q = 2.103x10^-2^). *CNN1*, *DES*, *CDC42EP2*, and *MYH11* were implicated in Supramolecular Fiber Organisation (GO:0097435, q = 2.029x10^-4^).

#### Pancreatic Insufficiency-Anaemia-Hyperostosis Syndrome

When the causal gene, *COX4I2*, was added to list 8^+^ (n = 32+1) there we no ontological associations made between list genes and the causal gene and was not considered further.

#### Megacystis-Microcolon-Intestinal Hypoperistalsis Syndrome

When the causal genes *MYL9*, *MYLK*, and *ACTG2* (n = 32+3, causal genes *LMOD1* and *MYH11* were already in list 8^+^) were added to list 8^+^ *SGCA* with at least one causal gene was associated with Muscle Contraction (GO:0006936, q = 1.758x10^-4^), Muscle Organ Development (GO:0007517, q = 6.9x10^-3^), Limb-girdle Muscle Atrophy (HP:0003797, q = 8.587x10^-3^), Abnormality of Calf Musculature (HP:0001430, q = 1.580x10^-2^), EMG: Myopathic Abnormalities (HP:0003458, q = 1.776x10^-2^), and Dilated Cardiomyopathy (q = 2.059x10^-2^). *CDC42EP2* with at least one causal gene was associated with Supramolecular Fiber Organisation (GO:0097435, q = 1.203x10^-3^), Positive Regulation of Cytoskeleton Organisation (GO:0051495, q = 1.054x10^-3^), Positive Regulation of Supramolecular Fiber Organisation (GO:1902905, q = 1.845x10^-2^), and Actin Filament Organisation (GO:0007015, q = 4.951x10^-2^). *HSPB7* with at least one causal gene was associated with Regulation of Blood Circulation (GO:1903522, q = 1.664x10^-3^) and Regulation of Heart Contraction (GO:0008016, q = 8.728x10^-3^). *SORBS1* with at least one causal gene was implicated through Actomyosin Structure Organisation (GO:0031032, q = 1.054x10^-3^). *FOXF1* with at least one causal gene was implicated through Duodenal Stenosis (HP:0100867, q = 1.776x10^-2^), Small Intestine Stenosis (HP:0012848, q = 1.776x10^-2^), Abnormality of the Duodenum (HP:0002246, q = 2.244x10^-2^), Intestinal Malrotation (HP:0002566, q = 3.521x10^-2^) and Hydronephrosis (HP:0000126, q = 3.521x10^-2^). *PNPLA2* with at least one causal gene was associated with Neck Muscle Weakness (HP:0000467, q = 1.776x10^-2^). *KCNMB1* with at least one causal gene was associated with Arterial Hypertension (q = 2.745x10^-2^).

#### Classical-like Ehlers-Danlos Syndrome type 1

When the causal gene, *TNXB*, was added to list 8^+^ (n = 32+1) *CDC42EP2* with *TNXB* was associated with Positive Regulation of Supramolecular Fiber Organisation (GO:1902905, q = 1.388x10^-3^) while *CDC42EP2* and *CNN1* were implicated through Supramolecular Fiber Organisation (GO:0097435, q = 1.388x10^-3^).

#### Retinoblastoma

When the causal gene, *RB1*, was added to list 6^-^ (n = 21+1) *REST* and *RB1* was associated with in Myoblast Differentiation (GO:0045445, q = 9.938x10^-3^), and *CHTOP* and *REST* with *RB1* Were associated with Chromatin Remodelling (GO:0006325, q = 3.517x10^-2^), and Chromatin Organisation (GO:0006325, q = 4.909x10^-2^). *RB1* and the overlap gene *TIA1* implicated *FXR1* and *REST* through Negative Regulation of Gene Expression (GO:0010629, q = 1.549x10^-2^).

#### Borjeson-Forssman-Lehmann Syndrome

When the causal gene, *PHF6*, was added to list 7^-^ (n = 39+1) no ontological associations were made between the non-overlapping list genes and the causal gene and was not considered further.

#### Lynch Syndrome

When the causal genes *EPCAM*, *MLH1*, *MLH3*, *MSH2*, *MSH6*, *PMS2*, and *TGFBR2* were added to list 7^-^ (n = 39+7) the gene *HLTF* and at least one causal gene was associated with DNA repair (GO:0006281, q = 4.066x10^-4^) and DNA Metabolic Process (GO:4.812x10^-2^). *CHTOP* with at least one causal gene was associated with Positive Regulation of ATP-dependent Activity (GO:0032781, q = 4.049x10^-3^) and Positive Regulation of Catalytic Activity (GO:0043085, q = 4.049x10^-3^). *WAPL* with at least one causal gene was associated with Negative Regulation of DNA metabolic Process (GO:0051053, q = 5.155x10^-3^), Protein Localisation to Chromatin (GO:0071168, q = 3.609x10^-2^), and Protein Localisation to Chromosome (GO:0033402, q = 4.812x10^-2^). *MTF2*, *ZMYM4*, and *ZNF146* with at least one causal gene were associated with DNA Binding (GO:003677, q = 1.848x10^-2^). *ADNP* with at least one causal gene was associated with the Joint hypermobility phenotype (HP:0001388, q = 2.712x10^-2^).

#### Literature associations

A literature search for previous associations made between the potentially novel genes and the causal gene(s), the potential role of O_2_^-^ and H_2_O_2_. Of the 31 positive list genes of interest 26 could be linked to the relevant 10 disorders, 3 genes had previously been associated, and 2 genes could not be associated. Of the 10 negative list genes of interest 9 could be linked to the disorders but 1 gene could not be associated through the literature.

## Discussion

This study investigated statistically significant associations between *SOD3* correlated genes and rare diseases using Pearson correlations, gene ontology, and phenotype ontology. The results support the Oxidative Stress Theory of Disease, and the hypothesis that *SOD3* correlated genes and oxidative stress play a role in disease and presentation should be accepted for the rare diseases considered.

No disorders were significant when interrogating the gene lists with the ClinVar database via Enrichr, however, several rare diseases were returned from the Orphanet database.

The list genes are predominantly found in smooth muscle cells and fibroblasts. *SOD3* positively correlated genes are primarily associated with cardiovascular and muscular disorders. It may be that increased respiration demanded by muscular inefficiencies results in elevated O_2_^-^, thus *SOD3* expression correlating with genes associated with muscular disorders. Negatively correlated genes were primarily associated with tumours. This may be due to angiogenesis lagging behind cancer growth, resulting in hypoxic new cells lacking the oxygen required to produce O_2_^-^ during respiration. Consequently, there is a decreased demand for SOD3, and *SOD3* expression negatively correlates with genes associated with cancer related disorders. As all the genes correlate with *SOD3* expression consideration of O_2_^-^ and H_2_O_2_ must be given. Oxidative stress can cause fibrosis, cardiovascular disorders, and other presentations in the disorders considered.

Diet may also play a role as high fat diets substantially increase H_2_O_2_ levels. H_2_O_2_ reacts with Cu^2+^ to produce HO_2_^-^ which oxidises cell membrane phospholipids (lipid peroxidation) thereby diminishing cell integrity and disturbing inter- and extra-cellular binding. This mechanism appears to be a factor in several of the disorders.

Many of the disorders also have systemic presentations and are diagnosed perinatally. Interestingly they present in tissue(s) arising from the mesodermal stem cell layer of the embryo where Transforming Growth Factor beta (TGF-β) is a key regulator of stem cell renewal and differentiation and its receptors are found in both fibroblasts and smooth muscle. Though the TGF-β genes Pearson correlations (ρ<0.25) fell outside criteria range it featured prominently enough in the literature and appears to play a role in several disorders to warrant consideration. TGF-β1 activates NOX enzymes thereby increasing H_2_O_2_ production. TGF-β also downregulates superoxide dismutase and glutathione peroxidase thereby increasing both O_2_^-^ and H_2_O_2_ induced oxidative stress. There is growing evidence to suggest SODs and TGF-β engage in reciprocal regulation. Research using rabbits with heart disease has shown that NOX4 interacts with Smad 2/3 to regulate TGF-β signalling thereby indirectly determining fibroblast to myofibroblast differentiation and inhibition of NOX4 was shown to have the potential as an efficacious therapy for heart disease [14]. Additionally, TGF-β promotes epithelial-to-mesenchymal trans-differentiation which is found in fibrosis and many cancers.

**Autosomal Dominant Cutis Laxa** is caused by a mutation in the *ELN*, *FBLN5*, or *ALDH18A1* genes. It affects fewer than one in a million people and presents as loose skin and associated symptoms vary but may include aortic aneurysm, arterial dilation, arterial tortuosity, bronchiectasis, cardiac valve anomalies, emphysema, diverticulosis, genital prolapse, hernia, and/or pulmonary arterial stenosis. It tends to be diagnosed soon after birth and there appears to be no effect on life expectancy due to early diagnosis and treatment.

Type 1 Autosomal Dominant Cutis Laxa is caused by a mutation in the *ELN* gene causing elastin dysfunction and elastogenesis hindrance. Type 2 is due to a mutation in the *FBLN5* gene which results in unstable anchoring of elastin to the cell membrane. Type 3 is due to a mutation in the *ALDH18A1* gene leading to elastin and collagen synthesis hindrance. This type is distinctive as it often presents with impaired cognition.

*ALDH18A1* mutations have previously been shown to cause autosomal dominant and recessive forms of Cutis Laxa. Another dehydrogenase, glucose-6-phosphate dehydrogenase, promotes methylation of MYH11 (myosin heavy chain eleven) [15]. It may be that ALDH18A1 has a similar effect on MYH11 which could explain the loss of mental capacity in Autosomal Dominant Cutis Laxa type 3 as MYH11 has previously been associated with neuronal disorders.

*MYH11* has been associated with vascular and connective tissue disorders. Mutations in the *MYH11* gene can result in aortic smooth muscle cell hyperplasia and stenosis and may upregulate *ACE* expression [16]. Increased MYH11 and LMOD1, and a lowering of O_2_^-^ have also been implicated in aortic vascular weakness. Intracranial stenosis has been associated with *MYH11* mutations [17] and under such conditions blood and oxygen supply is limited. Hypoxic neurons show increased oxidative stress further damaging neurons and encouraging cell death possibly resulting in diminished mental capacity. A link between *MYH11* and Cutis Laxa is not explicitly stated but may be inferred from a review of Loeys-Dietz Syndrome by Velchev *et al*. [18].

During cellular oxidative stress MYH11 is prone to oxidation which inhibits muscle contraction. O_2_^-^ induces downregulation of cGMP thus reduces cGMP-IRAG1-IP3 complex activity and downstream Ca^2+^ signalling / muscle contraction via heat shock protein family B (small) member 6 (HSPB6).

*AOC3* (amine oxidase copper containing 3) was implicated by causal genes *ELN* and *FBLN5* in age related macular degeneration which typically develops post-middle age, but it has previously been associated with Autosomal Dominant Cutis Laxa [19]. AOC3 is stored in intracellular vesicles which are released during the inflammatory response where it contributes to extracellular H_2_O_2_ production. Oxidative stress may contribute to the progression of macular degeneration by promoting lysosomal dysfunction within retinal pigment epithelial cells [20].

*AOC3* is involved in cardiac remodelling via its metabolite H_2_O_2_. The gene may contribute to the vascular anomalies and stenosis seen in this disorder. AOC3 uses a copper cofactor to catalyse amines into aldehydes which are the target of aldehyde dehydrogenases such as ALDH18A1. SOD3 also utilises a copper cofactor to catalyse O_2_^-^ into H_2_O_2_.

SOD3 binds with both collagen and causal gene product fibulin 5 (FBLN5) thus contributes to extracellular matrix stability. Failure of SOD3 binding to FBLN5 would have two notable consequences: crosslinking of collagen and fibulin may be compromised leading to matrix displacement, and displaced SOD3 could lead to increased local extracellular O_2_^-^. This O_2_^-^ increase could lead to protein damage and diffusion through aquaporins into smooth muscle cells leading to autophagy. O_2_^-^ and H_2_O_2_ increase calcium (Ca^2+^) permeability leading to dysregulation and endothelial dysfunction. Additionally, O_2_^-^ increases NO and decreases both cGMP and cAMP leading to muscle fibre relaxation.

*SOD3* is under-expressed in aortic aneurysms [21] thus extracellular O_2_^-^ scavenging is diminished. As extracellular O_2_^-^ increases extracellular H_2_O_2_ production decreases and the ROS can diffuse along concentration gradients in opposite directions via aquaporins. The resulting increase in intracellular O_2_^-^ and a decrease in H_2_O_2_ can lead to upregulation of *SOD1* to scavenge the excess O_2_^-^ and increase the H_2_O_2_ concentration. H_2_O_2_ inhibits myosin ATPase activity thus reducing actin binding and muscle contractility. The imbalance in both O_2_^-^ and H_2_O_2_ could result in muscle contraction and matrix instability, and may contribute to vascular smooth muscle weakness and skin laxity phenotypes. A review by Zhang *et al*. [22] also confirms O_2_^-^ as a causal component of skeletal muscle atrophy. H_2_O_2_ can modulate neuronal activity such as exciting GABAergic neurons and inhibiting dopaminergic neurons, it can also induce neuronal cell death. This may contribute to the symptoms of Autosomal Dominant Cutis Laxa type 3.

TGF-β has been found to reduce *ELN* mRNA recycling thereby increasing ELN concentration, though whether this is due to the increased in O_2_^-^/H_2_O_2_ oxidative stress remains unclear.

We propose that *AOC3* and *MYH11* are novel genes in Autosomal Dominant Cutis Laxa. *AOC3*, *MYH11*, superoxide, hydrogen peroxide, and copper should be further investigated as potential biomarkers. Further investigation into the potential role of TGF-β is also recommended.

**Lethal Arteriopathy Syndrome due to Fibulin-4 Deficiency** affects fewer than one in a million people and is caused by the *EFEMP2* (formerly *FBLN4*) gene. It presents with aneurysmal dilation, aortic/branch elongation, aortic/branch tortuosity, arachnodactyly, chest deformities, connective tissue dysfunction, hypertolerism, joint hypermobility, and pulmonary artery anomalies akin to those in the aorta along with potential stenosis. This disorder affects neonates and survival beyond infancy is uncommon.

EGF containing fibulin extracellular matrix protein 2 (*EFEMP2*) is essential for maintaining the integrity of the extracellular matrix around vascular smooth muscle, particularly surrounding the aorta. *EFEMP2* mutations have previously been identified as causing the aneurysmal aspects of the disease. However, a review by van de Luijtgaarden *et al*. [23] found no *EFEMP2* variants contributing to abdominal aortic aneurysm but noted *MYH11* mutations could result in an aneurysmal phenotype. The ambiguity over *EFEMP2* has since been clarified by *EFEMP2* silencing and knockdown experiments which confirmed the link between EFEMP2 and aortic aneurisms along with an increase in mesenchymal TAGLN [24]. This disorder is also known as Autosomal Recessive Cutis Laxa Type 1B (OMIM: 614437) as missense mutations in *EFEMP2* have also been shown to have impaired secretion and binding, and increased proteolysis leading to Cutis Laxa [25]. EFEMP2 and FBLN5 are prone to proteolysis, and both bind with ELN supporting its classification as a Cutis Laxa.

Mutations in *EFEMP2* can also affect TGF-β signalling leading to arterial aneurysms and tortuosity [26] as well as extracellular matrix synthesis and skeletal deformities [25]. Several of the symptoms, also seen in Loeys-Dietz Syndrome, are consistent with *TGFB2* mutations [27].

*MYH11* –see Autosomal Dominant Cutis Laxa (above)

A literature search returned no explicit connection between *SYNPO2* (Synaptopodin 2) and Cutis Laxa or Lethal Arteriopathy Syndrome due to Fibulin-4 Deficiency. *SYNPO2* contributes to actin filament formation in a calcium-dependant manner [28]. The effect of oxidative stress on Ca^2+^ is mentioned above (see Autosomal Dominant Cutis Laxa). SYNPO2 dimerization may also occur during oxidative stress [29] potentially weakening the protein chain between muscle fibre and matrix. Elevated H_2_O_2_ may lead to low-density lipoprotein peroxidation which has been implicated in fibrosis and a reduction in synaptopodin [30]. *SYNPO2* downregulation is associated with atherosclerosis and may contribute to the vascular anomalies and stenosis seen in this disorder.

We propose that *MYH11* and *SYNPO2* are novel genes in Lethal Arteriopathy Syndrome due to Fibulin-4 Deficiency. *MYH11*, *SYNPO2*, superoxide, and hydrogen peroxide, should be further investigated as potential biomarkers.

**EMILIN1-related Connective Tissue Disease** is caused by the *EMILIN1* gene and affects fewer than one in a million people. It typically presents with lower limb anomalies such as connective tissue defects, difficulty walking, foot deformities, hyperreflexia, joint hypermobility, motor neuropathy, muscle weakness and atrophy, skin hyper-elasticity, and tendon ruptures but may also present with aortic aneurysm and reduced intellectual capacity. This disorder can present in childhood but is usually diagnosed in adulthood and appears to have no effect on life expectancy. The disorder is also known as Distal Hereditary Motor Neuronopathy type X (OMIM: 620080) though the presentation is akin to Cutis Laxa and has been previously likened to type 1B [31]. Our research utilised the same overlapping genes and elicited the same novel genes for both EMILIN1-related Connective Tissue Disease and Lethal Arteriopathy Syndrome due to Fibulin-4 deficiency.

*MYH11*—see Autosomal Dominant Cutis Laxa (above)

*SYNPO2* intron sense-overlapping lncRNA is thought to regulate myogenesis and muscle atrophy. SYNPO2 may be implicated in the disorder by association. See Lethal Arteriopathy Syndrome due to Fibulin-4 Deficiency (above).

We propose that *MYH11* and *SYNPO2* are novel genes in EMILIN1-related Connective Tissue Disease with both contributing to presentations common to this disorder and Lethal Arteriopathy Syndrome due to Fibulin-4 Deficiency. *MYH11*, *SYNPO2*, *SYNPO2* intron sense-overlapping lncRNA, superoxide, and hydrogen peroxide should be investigated further as potential biomarkers.

**Renal Tubular Dysgenesis of Genetic Origin** is caused by mutations in *ACE*, *AGT*, *AGTR1*, or *REN* with unknown prevalence. It presents with proximal tubule hypoplasia, fetal anuria, oligohydramnios, Potter sequence traits, and pulmonary hypoplasia. This disorder develops in the fetus leading to death during the perinatal period usually due to kidney failure. Some treatments such as fludrocortisone and vasopressin appear to improve prognosis [32]. Renal transplant and dialysis may also improve life expectancy [33].

Gribouval *et al*. ([34] performed immunostaining to identify whether mutations in *REN* (Renin), then *ACE* (Angiotensin Converting Enzyme), *AGT* (angiotensinogen), and *AGTR1* (angiotensin II receptor type 1) were present in Renal Tubular Dysgenesis affected tissue hypothesizing all four genes were causal, and that dysfunction of any biomolecules involved in the renin-angiotensin system could potentially lead to Renal Tubular Dysgenesis. The involvement of the system is also indicated by correlations between renin-angiotensin system targeting drug use during pregnancy and Renal Tubular Dysgenesis development in the fetus. Disruption of the renin-angiotensin system leads to inadequate blood supply to developing fetal kidneys resulting in retarded renal tubular growth. Maternal hypertension or preeclampsia related hypertension could lead to the prescribing of Angiotensin Receptor Blockers to reduce blood pressure; however, these drugs reduce blood pressure in the hypotensive placenta and fetus thus contributes to the development of this disorder by further reducing nutrient supply. *SOD3* is also upregulated in the placenta of preeclampsia patients.

*TAGLN* (transgelin) was implicated by all four causal genes and the overlap gene *SOD3* in arterial hypertension.I Increased TAGLN in thoracic aneurysm has previously been reported by Senturk *et al*. [35]. *TAGLN* has also previously been associated with tubule related renal failure using a bioinformatics approach [36] and is upregulated in Passive Heymann nephritis [37]. *TAGLN* has low renal tubule expression under normal conditions, however, mouse models used to investigate nephropathic cystinosis revealed the upregulation of tubular *TAGLN* and increased O_2_^-^ was present in tubular swan neck lesions leading to apoptosis [38]. Conversely, H_2_O_2_ indirectly regulates muscle contraction by inhibiting heat shock protein family B (small) member 7 (HSPB7) promoting P13K/Akt pathway activity leading to inhibition of TAGLN [39]. H_2_O_2_ has also been reported as leading to insulin insufficiency [40] resulting in poor nutrient absorption further hindering tubule development. TGF-β upregulates *TAGLN* expression, possibly through increasing ROS production. SOD supplements have been found to have some efficacy in treating patients with chronic kidney disease by reducing TGF-β levels [41].

*AOC3* is discussed in Autosomal Dominant Cutis Laxa (above). AOC3 has previously been suggested as a biomarker in drug induced acute kidney injury [42].

Causal gene *ACE*, *AOC3*, and overlap gene *SOD3* were identified as significant in metal ion binding, and all three are copper binding. Cu^2+^ binds with angiotensin-I though the effect on functionality is poorly reported. Results from a study on silicosis suggest ACE and copper may be positively correlated, though this was not statistically analysed by the authors [43]. Copper has been associated with hypertension yet potential copper toxicity is rarely considered. A study investigating copper toxicity in yeast showed some ARBs can increase cellular copper tolerance [44], if this is translatable to humans it may be that ARBs mask symptoms of copper toxicity. This can cause renal tubular dysfunction and necrosis likely through copper induced oxidative stress and copper ion redox reactions.

Diet plays a key role in fetal development, especially during sensitive periods of tissue growth. Dietary sodium increases renal Nitric Oxide Synthase production [45] leading to elevated NO and O_2_^-^ levels. Yang *et al*. [46] showed that NO and O_2_^-^ can lead to upregulation of *COX2* in renal duct cells, a current and long-standing molecular target for analgesics. It is likely that this disorder is not only fatal, but painful, and there is a pressing need for screening and diagnostic biomarker discovery.

We propose that *AOC3* and *TAGLN* may be novel genes in the pathogenesis of Renal Tubular Dysgenesis of Genetic Origin. *AOC3*, *TAGLN*, superoxide, hydrogen peroxide, and copper should be further investigated as potential biomarkers.

**Holt-Oram Syndrome** is caused by the *TBX5* gene and has unknown prevalence. *TBX5* codes for T-Box Transcription Factor 5 which is associated with the expression of genes relating to heart and upper limb development. The disorder presents with limited upper limb growth with skeletal abnormalities such as triphalangeal thumb along with cardiac anomalies. Diagnosis tends to be given in childhood and it can lead to early demise.

No literature could be located associating *FOXF1* (forkhead box F1), *MYH11*, or *TAGLN* or their products with the disorder, however, oxidative stress could be a factor. Excessive cellular H_2_O_2_ can lead to *TBX5* promotor DNA damage in mouse embryonic stem cells leading to cardiac anomalies [47]. Mesoporous silica nanoparticles have shown some efficacy in relieving H_2_O_2_ stress in human embryonic stem cells and protected against H_2_O_2_ induced differentiation defects [48]. It would be reasonable to hypothesize glutathione peroxidase and over-the-counter reduced glutathione supplements may be similarly efficacious.

Following GWAS studies on Barrett’s Esophagus FOXF1 and TBX5 have been suggested as functionally related [49]. *FOXF1* was implicated in the disorder through cardiac anomaly related gene ontologies while *FOXF1* and *MYH11* were implicated through patent ductus arteriosus. Under-expression of *FOXF1* can cause lung abnormalities in mice [50]. Pulmonary symptoms such as Horseshoe Lung are rare but have presented in Holt-Oram Syndrome [51]. *FOXF1* dysregulation or mutation may be present in patients with Holt-Oram Syndrome, particularly those with pulmonary anomalies. Mutations in *FOX1* can also cause fetal cardiovascular anomalies [52]. The TGF-β1/Smad3 signalling pathway in cardiac fibroblasts can lead to fibrosis which may play a role in the disorder. It has been found that FOXF1 inhibits this pathway and can reduce progression of fibrosis [53].

TBX5 binds with *MYH11* intron 3 and deletion of the MYH11 binding site is associated with dysregulation of smooth muscle genes in the fetal heart [54]. *MYH11* mutations are associated with Patent Ductus Arteriosus [55], aortic dissection, and aortic aneurysms [56].

*MYH11*, *SOD3*, and *TAGLN* are all downregulated in Aortic Dissection [57]. No literature was located directly linking *TAGLN* and *TBX5*, however, *TAGLN* is upregulated during osteoblast differentiation of skeleto-stromal stem cells [58]. Dysregulation or mutations may contribute to symptoms of upper limb malformation.

We propose that *FOXF1*, *MYH11*, *and TAGLN* are novel genes in Holt-Oram Syndrome. *FOXF1*, *MYH11*, *TAGLN*, superoxide, and hydrogen peroxide should be investigated further as potential biomarkers. Further investigation into the potential role of TGF-β is also recommended.

**Multisystemic Smooth Muscle Dysfunction Syndrome** is caused by a mutation in the *ACTA2* gene and affects fewer than one in a million people. It presents with aortic anomalies, cerebrovascular artery restriction, intestinal hypoperistalsis, hypotonic bladder, pulmonary artery hypertension, unresponsive dilated pupils, patent ductus arteriosus, mydriasis, aneurysms, and intestinal malrotation.

*MYH11* mutations have previously been associated with Megacystis-Microcolon-Intestinal Hypoperistalsis Syndrome, however, Yetman and Starr [59] describe a case where an *MYH11* frameshift mutation was present in a suspected Multisystemic Smooth Muscle Dysfunction Syndrome / Megacystis-Microcolon-Intestinal Hypoperistalsis Syndrome patient without the causal *ACTA2* gene mutations, proposing a novel recessive *MYH11* presentation. Other case reports have identified *MYH11* mutations in chronic intestinal pseudo-obstruction and Pseudoileus. TGF-β1 is implicated by promoting *MYH11* expression.

Post-translational modifications to ACTA2 regulate actin-myosin binding and thus smooth muscle contraction. This functional relationship explains the phenotypic similarities between Multisystemic Smooth Muscle Dysfunction Syndrome caused by *ACTA2* mutations, and Megacystis-Microcolon-Intestinal Hypoperistalsis Syndrome 2 caused by *MYH11* mutations.

*FOXF1* downregulation has been implicated in intestinal pseudo-obstruction [60] though research is lacking. Sense strand *FOXF1* transcription is concurrent with antisense *FOXF1* adjacent non-coding developmental regulatory RNA (FENDRR). FENDRR increases *ACTA2* expression and the risk of cardiac fibrosis [61]. It could be that mutations effecting FOXF1 or FENDRR could reduce ACTA2 activity resulting in cellular dysfunction akin to that seen in Multisystemic Smooth Muscle Dysfunction Syndrome.

No literature was located directly linking *KCNMB1* (potassium calcium-activated channel subfamily M regulatory beta subunit 1) to Multisystemic Smooth Muscle Dysfunction Syndrome. Oxidative stress can lead to O_2_^-^ and H_2_O_2_ induced increase in Ca^2+^ membrane permeability as described above (see Autosomal Dominant Cutis Laxa). KCNMB1 is a regulatory subunit of KCNMA1 which modulates KCNMA1 Ca^2+^ sensitivity. An increase in Ca^2+^ activates the channel for the export of potassium (K^+^). Dysregulation of membrane potential due to dysfunction of KCNMB1 could lead to smooth muscle contraction dysfunction.

No literature was located directly linking *PLN* (phospholamban) to Multisystemic Smooth Muscle Dysfunction Syndrome. PLN inhibits sarcoplasmic reticulum Ca^2+^-ATPase thus regulates intracellular Ca^2+^ concentration and muscle contraction. As O_2_^-^ decreases cAMP, cAMP-dependent protein kinase phosphorylation of PLN is limited leading to Ca^2+^ dysregulation and muscle dysfunction. Dilated cardiomyopathy can occur if PLN excessively inhibits the calcium pump of the sarcoplasmic reticulum [62]. PLN has been shown to be upregulated by TGF-β1 in smooth muscle [63].

We propose that *FOXF1*, *KCNMB1*, *and MYH11* are novel genes in Multisystemic Smooth Muscle Dysfunction Syndrome. *FOXF1*, *KCNMB1*, *MYH11*, superoxide, and hydrogen peroxide should be investigated further as potential biomarkers. Further investigation into the potential role of TGF-β is also recommended.

**Distal Hereditary Motor Neuropathy type 2** can be caused by any of four genes: *HSPB8*, *HSPB1*, *HSPB3*, or *FBXO38* and affects fewer than one in a million people. It presents with progressive peripheral neuropathy, and motor weakness in the lower extremities and can lead to paralysis. Diagnosis tends to occur during adulthood and appears to have no impact upon life expectancy.

No literature could be located which suggested interactions between any of the causal genes and the ontology identified non-overlapping list genes linked with the disorder in this research.

TAGLN regulates actin-myosin motor function and may contribute to the disorder during oxidative stress states (see above: Renal Tubular Dysgenesis of Genetic Origin). *TAGLN* which is mainly expressed in smooth muscle cells is also differentially expressed in sensory neurons and differentiating mesenchymal cells [64]. The same research also showed a glutathione peroxidase precursor to be differentially expressed in sensory neurons, suggesting H_2_O_2_ may play a role in the disorder.

*PNPLA2* (patatin like phospholipase domain containing 2) is essential for the hydrolysis of lipid droplet triglycerides. PNPLA2 regulates lipolytic energy release required for muscle fibre function with dysregulation leading to motor deficiency in mice [65]. Mutations in *PNPLA2* can cause Neutral Lipid Storage Disease with Myopathy (OMIM: 610717) which presents with similar symptoms suggesting both disorders share a common pathway via PNPLA2. PNPLA2 dysfunction can cause oxidative stress in myoblasts in mice, but this is ameliorated with N-acetylcysteine treatment [66]. The rescue mechanism is believed to involve N-acetylcysteine, a cysteine prodrug, being catalysed into glutathione precursors resulting in elevated glutathione peroxidase, thereby aiding H_2_O_2_ scavenging and a reduction in oxidative stress [67].

For how MYH11 reacts to oxidative stress see above (Autosomal Dominant Cutis Laxa).

See Autosomal Dominant Cutis Laxa and Multisystemic Smooth Muscle Dysfunction Syndrome above for information on *KCNMB1*.

We propose that *KCNMB1*, *MYH11*, *TAGLN*, *and PNPLA2* are novel genes in Distal Hereditary Motor Neuropathy type 2. *KCNMB1*, *MYH11*, *TAGLN*, *PNPLA2*, superoxide, and hydrogen peroxide should be investigated further as potential biomarkers.

**Congenital Glaucoma** can be caused by the *CYP1B1*, *LTBP2*, *TEK*, and two loci of uncertain function which were not investigated in this research. It affects between one in a hundred thousand and one in ten thousand people. It presents with increased eye and corneal size, elevated intraocular pressure, corneal edema, Haab striae, and optic nerve cupping leading to blindness. It tends to be diagnosed in infants and appears to have no impact upon life expectancy.

*CLDN5* (claudin 5) could not be directly linked to the causal genes through a literature search, and little could be found to connect *CLDN5* to glaucoma, however, a case study by Cordovez *et al*. [68] found *CLDN5* duplication to be a potential cause of Congenital Glaucoma. The mechanics of this is yet to be elucidated though angular aqueous plexus cells are associated with porcine glaucoma and oxidative stress within these cells can lead to upregulation of *CLDN5* [69]. O_2_^-^ can combine with NO from nitric oxide synthases to form peroxynitrite (NO_3_^-^) which has been shown to reduce *CLDN5* expression [70]. *CLDN5* expression appears not to be pressure dependent, however, TGF-β1 reduces CLDN5 expression which contributes to the loosening of the blood brain barrier [71] and increase oxidative stress [72].

*KCNMB1* in Schlemm’s canal cells contribute to the outflow pathway thus is a regulator of intraocular pressure and may contribute to glaucoma [73]. See Multisystemic Smooth Muscle Dysfunction Syndrome above for information regarding oxidative stress conditions.

The trabecular meshwork controls outflow and intraocular pressure and anomalies in the cytoskeleton of these cells are believed to be a cause of glaucoma. The *CDC42EP2* (CDC42 effector protein 2) product has previously been associated with cytoskeletal organisation andglaucoma.

The literature did not reveal any previous association between CNN1 (calponin 1) and glaucoma, however, TGF-β1 promotes *CNN1* expression and CNN1 has been identified in the trabecular meshwork [74].

DES (Desmin) has not been detected in the trabecular meshwork, however, DES has been found in fibroblasts [75], ciliary muscle, and iris dilator muscle [76]. DES containing myofibroblasts can develop following epithelial-stromal injury which in turn can lead to corneal opacity and fibrosis [77] but activation of TGF-β can trigger myofibroblast apoptosis [78].

Literature searches failed to find a direct link between *MYH11* and glaucoma. It may be that *MYH11* has an unknown influence upon glaucoma, possibly through vascular or ciliary body dysfunction.

CYP1B1 deficiency can lead to H_2_O_2_ induced oxidative stress and lipid peroxidation in the trabecular meshwork and is ameliorated by administration of N-acetylcysteine [79]. When the integrity of the trabecular meshwork is compromised outflow is impeded leading to glaucoma [80].

We propose that *CNN1*, *and DES* are novel genes in Congenital Glaucoma. *CNN1*, *DES*, superoxide and hydrogen peroxide should be further investigated as potential biomarkers.

**Pancreatic Insufficiency-Anemia-Hyperostosis Syndrome** failed to be associated with non-overlapping list genes and no links were made to the causal gene, *COX4I2*, through gene ontology or a literature search.

**Megacystis-Microcolon-Intestinal Hypoperistalsis Syndrome** has five types caused by *MYLK*, *MYH11*, *LMOD1*, *MYL9*, and *ACTG2* genes and has unknown prevalence. The five types have clinical variability presenting with a range of symptoms including bladder smooth muscle dysfunction, dilation of the bladder, functional obstruction of the intestine, intestinal malrotation / volvulus, intestinal smooth muscle dysfunction, malnutrition, megacystis, multiple organ failure, neonatal functional gastrointestinal obstruction, prenatal bladder enlargement, secondary hydronephrosis, sepsis, dependency upon total parenteral nutrition. Diagnosis tends to be neonatal or during infancy. Type 2 shows a female bias. Types 2 and 3 are often fatal but some treatments and surgeries have shown limited success.

*LMOD1* and *MYH11* are causal genes which appeared in the *SOD3* correlation lists. None of the non-overlapping list genes could be directly linked to Megacystis-Microcolon-Intestinal Hypoperistalsis Syndrome through a literature search.

*SGCA* (sarcoglycan alpha) has been associated with muscular dystrophies. Cyanidin which is found in many berries acts as an O_2_^-^ scavenger and appears to slow progression of muscular dystrophies in SGCA deficient mice [81] suggesting oxidative stress involvement.

Both *CDC42* and *SGCA* are downregulated in muscle atrophy [82]. CDC42-binding kinases can phosphorylate MYL9 linking *CDC42EP2* to the causal gene *MYL9* in muscle contraction. CDC42 contributes to O_2_^-^ production via NOX enzymes. CDC42 has also been suggested as a therapeutic target for renal fibrosis induced by TGF-β [83].

For the influence of *FOXF1* see Multisystemic Smooth Muscle Dysfunction Syndrome.

Myocardin is involved in smooth muscle differentiation and *SORBS1* (sorbin and SH3 domain containing 1) has previously been associated with Myocardin using a bioinformatics / correlation approach [84]. Decreases in SORBS1 can lead to under-expressed superoxide dismutases and oxidative stress in umbilical vein endothelial cells [85].

We propose that *CDC42EP2*, *FOXF1*, *SGCA*, *and SORBS1* are novel genes in Megacystis-Microcolon-Intestinal Hypoperistalsis Syndrome. *CDC42EP2*, *FOXF1*, *SGCA*, *SORBS1*, superoxide, and hydrogen peroxide should be further investigated as potential biomarkers.

**Classical-like Ehlers-Danlos Syndrome type 1** is caused by the *TNXB* gene and affects less than one in a million people. It presents with joint hypermobility, skin hyperextensibility, easy bruising, foot and hand deformities, severe fatigue, muscle weakness, myalgia, and tissue fragility (e.g., blood vessel fragility). Diagnosis tends to be pre-adulthood, often during infancy, and does not appear to affect life expectancy.

No direct link between *CNN1* or *CDC42EP2* could be made with *TNXB* or Classical-like Ehlers-Danlos Syndrome (cEDS) through a literature search. However, there have been reports of atherosclerotic presentations and CNN1 and the TGF-β pathway both contribute to atherosclerotic matrix ‘stiffness’ [86] which may be a contributing factor.

We propose that *CNN1* is a novel gene in Classical-like Ehlers-Danlos Syndrome type 1. *CNN1* should be further investigated as a potential biomarker. Further investigation into the potential role of TGF-β is also recommended. In S3 File, we present an example of experimental case supporting CNN1 as a novel gene in cEDS.

**Retinoblastoma** is caused by the *RB1* gene and has unknown prevalence. It has a childhood bias and is often fatal but can, in some cases, be successfully treated.

*FXR1* (FMR1 autosomal homolog 1) has previously been implicated in cancers such as hepatocellular carcinoma, colorectal cancer, and urothelial carcinoma [87]. FXR1 also indirectly regulates TGF-β activity [88].

*REST* (RE1 silencing transcription factor) and *RB1* expressions are both downregulated by Interleukin 6 in cancer cells [89]. Interleukin 6 also upregulates *SOD2* leading to increased H_2_O_2_ production [90]. Elevated H_2_O_2_ also leads to STAT3 activation [91] which is a factor in Retinoblastoma and potential therapeutic target [92]. REST reduces H_2_O_2_ induced oxidative stress thereby restoring oxidative balance. REST and TGF-β are also upregulated in cytotoxic T lymphocytes in hepatic tumour [93].

The influence of *CHTOP* (chromatin target of PRMT1) in cancerous cells is severely lacking research. Methylation of CHTOP promotes 5FMC recruitment, this complex then interacts with the methylosome to regulate the expression of glioblastoma promoting genes [94]. This may translate to Retinoblastoma. While PMRT1 appears to have no interaction with RB1, PMRT1 and TGF-β are mutually regulatory thus may indirectly influence retinoblastoma presentation through ROS management.

We propose that *CHTOP*, *FXR1*, *and REST* are novel genes in Retinoblastoma. *CHTOP*, *FXR1*, and *REST*, should be investigated further as potential biomarkers. Further investigation into the potential role of TGF-β is also recommended.

**Borjeson-Forssman-Lehmann Syndrome** had no new genes to investigate.

**Lynch Syndrome** can be caused by *EPCAM*, *MLH1*, *MLH3*, *MSH2*, *MSH6*, *PMS2*, or *TGFBR2* genes and prevalence are unknown. It may lead to a variety of cancers including those of the gastrointestinal tract, urinary tract, kidneys, endometrium, ovary, brain, prostate, and skin. Age of diagnosis and prognosis is variable.

HLTF (helicase like transcription factor) and MSH2 interact during DNA mismatch repair and HLTF is also involved in nucleotide excision repair with the NER incision complex [95]. HLTF has been associated with several cancers including colorectal cancer [96], head and neck cancer [97], Chronic Myeloid Leukemia [98]. TGF-β upregulates HLTF which attenuates migration of colorectal cancer cells by hindering TGF-β/Smad signalling [99].

WAPL (WAPL cohesin release factor) competes with Sororin and PD-L1 for cohesin complex binding with WAPL thereby regulating chromosome cohesion, local gene expression, and cell proliferation [100, 101]. WAPL contributes to DNA repair thus safeguards against cohesion loss and dysregulation of oncogene expression [102]. However, overexpressed *WAPL* can also lead to chromosome destabilisation [103]. Additionally, Menin-associated proteins such as WAPL play a role in maintaining sufficient cellular energy metabolism to maintain cancer growth [104]. WAPL has been associated with several cancers such as cervical cancer and esophageal carcinoma.

*MTF2* expression is correlated with age and is differentially expressed in thirty cancers [105]. *MTF2* is upregulated in colorectal carcinoma (commonly arising from Lynch Syndrome) and contributes to copper homeostasis which is dysregulated in many cancers [106]. MTF2 has been suggested as a therapeutic target for myeloma and Acute Myeloid Leukemia. Interestingly, Retinoblastoma (see above) develops utilising the lncRNA CCAT1/miR-218-5p/MTF2 axis [107].

The literature revealed little regarding *ZMYM4* (zinc finger MYM-type containing 4). The *ZMYM4-OPRD1* fusion gene has been associated with breast cancer [108], while ZMYM4 has been associated with colonic and gastric cancers, malignant melanoma, hepatocellular carcinoma and pancreatic carcinomas.

*ADNP* is important for cell signalling and cancer growth and has been suggested as a potential as a biomarker and / or therapeutic target for gastric and colorectal cancer, hepatocellular carcinoma, glioblastoma multiforme, Bladder cancer, ovarian cancer, and breast cancer. ADNP may influence cancer cell migration through the TGF-β /Smad signalling [109].

No link to Lynch Syndrome could be made for *ZNF146* (zinc finger protein 146) through a literature search.

See above (Retinoblastoma) for information on *CHTOP*.

Interestingly, while *SOD3* is differentially expressed in many cancers, no link between oxidative stress and Lynch Syndrome associated genes could be made.

We propose that *ADNP*, *CHTOP*, *HLTF*, *MTF2*, *WAPL*, *and ZMYM4* are novel genes in Lynch Syndrome. *ADNP*, *CHTOP*, *HLTF*, *MTF2*, *WAPL*, and *ZMYM4*, should be further investigated as potential biomarkers. Further investigation into the potential role of TGF-β is also recommended.

## Limitations

This research relies on previously reported research and data which may in some cases be anomalous leading to erroneous interpretations. Experimental verification of the hypothesized pathways is recommended. The method assumes genes currently believed to be causal are the only causal genes and several contributing pathways may not have been identified. Additionally, other ontology and pathway collections could have been utilised giving variation to the results and hypotheses.

Online databases are imperfect with an estimated data input error rate of between 2.3% and 26.9% [110]. Data extraction errors also occur in research, and this may be as high as 50% in systematic reviews [111], and while the data given in this research was correct at the time of study this data may change due to database updates. Disorders were excluded despite having significant overlap with the correlate gene lists and readers are encouraged to consider the lists uploaded to Enrichr. While citations in this research are peer reviewed the quality of the cited research may vary. The authors are also not infallible to input and interpretation errors. Software and programming may at some point be determined as sub-optimal. RMA normalisation could have produced artefact genes not associated with *SOD3* and while the discussion highlights gene / product interactions with previous experimental evidence it is possible that some results are anomalous or coincidental. An unlikely but possible and little considered source of errors in computational research are ‘single event upsets’ causing small but potentially cumulative and significant data corruption.

Due to financial limitations only openly available data sources were used, and the quality likely lags paywalled databases. Similarly, only open source and freeware software was used which limited the efficiency of computational analysis. Time limitations restricted the literature search due to the many aliases and former names of genes. *HSPB6*, *LMOD1*, and *SOD3* were overlapping genes in all disorders discussed, however, this was not investigated and further research into how these genes and their products may interact is recommended.

Long non-coding RNA probes were excluded; in hindsight some valuable insights may have been missed. This research was authored by two bioinformatics researchers without the backing of a team of experts in the appropriate fields of rare diseases and could be expanded upon in various directions, and there is potential for unchecked unconscious bias.

## Conclusion

From a list of 100 genes statistically correlated with *SOD3*, 40 (22 unique) were ontologically associated with 12 significantly overlapping rare disorders. 35 of those genes (21 unique) are proffered as novel. Utilising peer reviewed literature novel gene mechanisms contributing to symptomatic traits were suggested for: Autosomal Dominant Cutis Laxa (*AOC3* and *MYH11*), Lethal Arteriopathy Syndrome due to Fibulin-4 Deficiency (*AOC3* and *SYNPO2*), EMILIN1-related Connective Tissue Disease (*MYH11* and *SYNPO2*), Renal Tubular Dysgenesis of Genetic Origin (*AOC3* and *TAGLN*), Holt-Oram Syndrome (*FOXF1*, *MYH11*, and *TAGLN*), Multisystemic Smooth Muscle Dysfunction Syndrome (*FOXF1*, *KCNMB1*, *MYH11*, *PLN*), Distal Hereditary Motor Neuropathy Syndrome type 2 (*KCNMB1*, *MYH11*, *TAGLN*, and *PNPLA2*), Congenital Glaucoma (*CNN1* and *DES*), Megacystis-Microcolon-Intestinal Hypoperistalsis Syndrome (*CDC42EP2*, *FOXF1*, *SGCA*, and *SORBS1*), Classical-like Ehlers-Danlos Syndrome type 1 (*CNN1*), Retinoblastoma (*CHTOP*, *FXR1*, and *REST*), and Lynch Syndrome (*ADNP*, *CHTOP*, *HTLF*, *MTF2*, *WAPL*, and *ZMYM4*). These genes are suggested as potential biomarkers for the relevant disorders.

*SOD3* was inherently an overlapping gene previously associated with the positively correlated genes and oxidative stress was found to play a role in 10 out of 12 disorders (83%). The two disorders oxidative stress could not be linked to were Classical-like Ehlers-Danlos Syndrome type 1 and Lynch Syndrome.

*SOD3* expression diminishes with age leading to increased risk of O_2_^-^ induced oxidative stress associated disorders and high fat diets contribute to H_2_O_2_ production. Further dietary studies into the effects of oxidative stress are required to determine biomarker potential for the disorders discussed.

Lethal Arteriopathy Syndrome due to Fibulin-4 Deficiency and EMILIN-1 Related Connective Tissue Disease are closely related in terms of genetic aetiology and perhaps should be reclassified as Cutis Laxa. It may be that Loeys-Dietz Syndrome and Ehlers–Danlos Syndrome also share a pathway with the disorders thus it may be prudent to consider the disorders collectively when new findings for one arise.

Several of the disorders develop pre-birth and TGF-β was implicated in 10 of the 12 disorders (83%), the two without TGF-β association were Renal Tubular Dysgenesis of Genetic Origin and Distal Hereditary Motor Neuropathy type 2. TGF-β is essential for development, being particularly active in the mesodermal layer of the embryo. Studies investigating how embryonic oxidative stress may influence phenotypes via TGF-β are recommended, and the role of TGF-β in the disorders discussed should be investigated further.

While superoxide dismutase and reduced glutathione are available as low-cost supplements with safe ADMET profiles the therapeutic efficacy has not been widely studied. However, it is worth noting that SOD supplements, N-acetylcysteine, cyaniding, and Mesoporous silica nanoparticles have shown some efficacy in oxidative stress related disorders. Superoxide dismutase, glutathione peroxidase, and the genes identified within this research are proffered as potential biomarkers and therapeutic targets in the rare diseases discussed.

Copper may also play a role in the presentation of rare diseases and are suggested as potential biomarkers for the disorders discussed, particularly Autosomal Dominant Cutis Laxa and Renal Tubular Dysgenesis of Genetic Origin.

## Supporting information

S1 File Formulae and coding. (DOCX)

S2 File Gene lists. (DOCX)

S3 File Experimental case supporting CNN1 as a novel gene in cEDS. (DOCX)
