# Supplementary material for: Elucidating the roles of SOD3 correlated genes and reactive oxygen species in rare human diseases using a bioinformatic-ontology approach
Source: PLoS One. 2024 Oct 31;19(10):e0313139. doi: 10.1371/journal.pone.0313139 (PMC11527182; doi:10.1371/journal.pone.0313139)
Supplement: S1 File — (DOCX) [file pone.0313139.s001.docx]

# Supplementary file 1: Formulae and coding

**Microsoft Excel 365 formulae**

Pearson Correlation (*rho*): =PEARSON(X_1_:X_2_,Y_1_:Y_2_)

Two-tail t-test statistic (*tstat*): =*rho**SQRT(n-2)/SQRT(1-*rho*^2)

Two-tail t-test p-value: =T.DIST.2T(ABS(*tstat*),n-2)

Jarque-Bera Test statistic (*JBTS*): =(COUNT(X_1_:X_2_)/6)*( SKEW(X_1_:X_2_) ^2+(KURT(X_1_:X_2_)^2-3)/4)

χ^2^ p-value for *JBTS:* =CHISQ.DIST.RT(abs(*JBTS*),2)

**RMA normalisation (R programming)**

Library(Affy)

raw.data <- ReadAffy(celfile.path = "GSE2109_RAW/")

normalised.data <- rma(raw.data)

normalised.expression <- as.data.frame(exprs(normalised.data))

write.table(normalised.expression, file = "GSE2109_normalised.txt", quote = FALSE, sep ="\t" ,row.names = TRUE, col.names = TRUE)

**Gene list robustness**

Library(pvclust)

data <- as.matrix(read.table("GSE2109_rho_34_CLEAN.txt", header=TRUE,row.names=1))

robustness<-pvclust((as.matrix(t(data))),method.dist="correlation", use.cor="pairwise.complete.obs", method.hclust="ward.D2",nboot=1000)

plot(robustness, hang=-1,cex=0.5, main="GSE2109 Correlation (|ρ|≥0.34) Cluster with p-values (%)")

pvrect(result,alpha=0.95)

**# Best fit from duplicates**

library(jetset)

jscores('hgu133plus2', symbol = 'CAND1')

jmap('hgu133plus2', symbol = "CAND1")

jscores('hgu133plus2', symbol = 'FBXO28')

jmap('hgu133plus2', symbol = "FBXO28")

jscores('hgu133plus2', symbol = 'HSPB6')

jmap('hgu133plus2', symbol = "HSPB6")

jscores('hgu133plus2', symbol = 'MREG')

jmap('hgu133plus2', symbol = "MREG")

jscores('hgu133plus2', symbol = 'MTF2')

jmap('hgu133plus2', symbol = "MTF2")

jscores('hgu133plus2', symbol = 'MYH11')

jmap('hgu133plus2', symbol = "MYH11")

jscores('hgu133plus2', symbol = 'PLN')

jmap('hgu133plus2', symbol = "PLN")

jscores('hgu133plus2', symbol = 'QSER1')

jmap('hgu133plus2', symbol = "QSER1")
