# Supplementary material for: Elucidating the roles of SOD3 correlated genes and reactive oxygen species in rare human diseases using a bioinformatic-ontology approach
Source: PLoS One. 2024 Oct 31;19(10):e0313139. doi: 10.1371/journal.pone.0313139 (PMC11527182; doi:10.1371/journal.pone.0313139)
Supplement: S2 File — (DOCX) [file pone.0313139.s002.docx]

# Supplementary file 2: Gene lists

**Table S1: List 1 SOD3 Pearson correlated genes ρ≥|0.41| (n=5).** List 1^+^ is also available at <https://maayanlab.cloud/Enrichr/enrich?dataset=5af529f562408af9c145d370be27bb82>.

| **1^+^ (n=5)** | **1^-^ (n=0)** |
| --- | --- |
| *HSPB6* | - |
| *HSPB7* | - |
| *MYH11* | - |
| *SOD3* | - |
| *TAGLN* | - |

**Table S2: List 2 SOD3 Pearson correlated genes ρ≥|0.40| (n=9).** List 2^+^ is also available at <https://maayanlab.cloud/Enrichr/enrich?dataset=934f821855a853ab005881d47c9681ae>.

| **2^+^ (n=9)** | **2^-^ (n=0)** |
| --- | --- |
| *AOC3* | - |
| *HSPB6* | - |
| *HSPB7* | - |
| *IRAG1* | - |
| *LMOD1* | - |
| *MYH11* | - |
| *SOD3* | - |
| *SYNPO2* | - |
| *TAGLN* | - |

**Table S3: List 3 SOD3 Pearson correlated genes ρ≥|0.39| (n=13).** List 3^+^ is also available at <https://maayanlab.cloud/Enrichr/enrich?dataset=c6f692d9b682f851604b581916a19740>, list 3^-^ is available at <https://maayanlab.cloud/Enrichr/enrich?dataset=d43eb1b7060338b81642cd58b14ee9e4>.

| **3^+^ (n=11)** | **3^-^ (n=2)** |
| --- | --- |
| *AOC3* | BTF3L4 |
| *CHRDL2* | CAND1 |
| *HSPB6* | - |
| *HSPB7* | - |
| *IRAG1* | - |
| *LIMS2* | - |
| *LMOD1* | - |
| *MYH11* | - |
| *SOD3* | - |
| *SYNPO2* | - |
| *TAGLN* | - |

**Table S4: List 4 SOD3 Pearson correlated genes ρ≥|0.38| (n=20).** List 4^+^ is also available at <https://maayanlab.cloud/Enrichr/enrich?dataset=454d160a7bc9403da11803236bda6cc7>, list 4^-^ is available at <https://maayanlab.cloud/Enrichr/enrich?dataset=45be7f05aefc552e8936fa36a17af693>.

| **4^+^ (n=13)** | **4^-^ (n=7)** |
| --- | --- |
| *AOC3* | *BTF3L4* |
| *CHRDL2* | *CAND1* |
| *CNN1* | *CTDSPL2* |
| *FOXF1* | *MREG* |
| *HSPB6* | *MSH2* |
| *HSPB7* | *RBM8A* |
| *IRAG1* | *TIA1* |
| *LIMS2* | - |
| *LMOD1* | - |
| *MYH11* | - |
| *SOD3* | - |
| *SYNPO2* | - |
| *TAGLN* | - |

**Table S5: List 5 SOD3 Pearson correlated genes ρ≥|0.37| (n=34).** List 5^+^ is also available at <https://maayanlab.cloud/Enrichr/enrich?dataset=bbf52fe9c6ed275533f86d4355c0e92a>, list 5^-^ is available at <https://maayanlab.cloud/Enrichr/enrich?dataset=38c541358f76eebd14c363e4ca50174b>.

| **5^+^ (n=19)** | **5^-^ (n=15)** |
| --- | --- |
| *AOC3* | *BTF3L4* |
| *CHRDL2* | *CAND1* |
| *CNN1* | *CNOT6* |
| *DES* | *CTDSPL2* |
| *FOXF1* | *FXR1* |
| *GPBAR1* | *KANSL2* |
| *HSPB6* | *MREG* |
| *HSPB7* | *MSH2* |
| *IRAG1* | *QSER1* |
| *KCNMB1* | *RBM8A* |
| *LIMS2* | *REST* |
| *LMOD1* | *TIA1* |
| *MYH11* | *XPO1* |
| *PLN* | *ZMYM4* |
| *PNPLA2* | *ZNF146* |
| *SGCA* | - |
| *SOD3* | - |
| *SYNPO2* | - |
| *TAGLN* | - |

**Table S6: List 6 SOD3 Pearson correlated genes ρ≥|0.36| (n=42).** List 6^+^ is also available at <https://maayanlab.cloud/Enrichr/enrich?dataset=ff86f50dfcf9ec84ce4908841808ce98>, list 6^-^ is available at <https://maayanlab.cloud/Enrichr/enrich?dataset=7394636a36bb5dd573dc71a569f133a7>.

| **6^+^ (n=21)** | **6^-^ (n=21)** |
| --- | --- |
| *AOC3* | *BTF3L4* |
| *CHRDL2* | *CAND1* |
| *CNN1* | *CHTOP* |
| *DES* | *CNOT6* |
| *FOXF1* | *CTDSPL2* |
| *GPBAR1* | *FXR1* |
| *HSPB6* | *G2E3* |
| *HSPB7* | *HAUS3* |
| *IRAG1* | *KANSL2* |
| *KCNMB1* | *MREG* |
| *LIMS2* | *MSH2* |
| *LMOD1* | *QSER1* |
| *MYH11* | *RBM8A* |
| *PLN* | *REST* |
| *PNPLA2* | *RPAP3* |
| *RILP* | *TIA1* |
| *SGCA* | *XPO1* |
| *SOD3* | *ZMYM4* |
| *SORBS1* | *ZNF107* |
| *SYNPO2* | *ZNF146* |
| *TAGLN* | *ZNF260* |

**Table S7: List 7 SOD3 Pearson correlated genes ρ≥|0.35| (n=68).** List 7^+^ is also available at <https://maayanlab.cloud/Enrichr/enrich?dataset=2db950edb668ce5b06800963f2a5f815>, list 7^-^ is available at <https://maayanlab.cloud/Enrichr/enrich?dataset=a01aeddaaa905797223806061ebb3ceb>.

| **7^+^ (n=29)** | **7^-^ (n=39)** |
| --- | --- |
| *AOC3* | *ADNP* |
| *CDC42EP2* | *BRWD1* |
| *CHRDL2* | *BTF3L4* |
| *CLDN5* | *CAND1* |
| *CNN1* | *CHTOP* |
| *DES* | *CNOT6* |
| *FOXF1* | *CTDSPL2* |
| *GPBAR1* | *DYNLT2B* |
| *HMCN2* | *FBXO28* |
| *HSPB6* | *FRMD8* |
| *HSPB7* | *FXR1* |
| *IRAG1* | *G2E3* |
| *KCNMB1* | *HAUS3* |
| *LIMS2* | *HLTF* |
| *LMOD1* | *HNRNPC* |
| *MUC3B* | *KANSL2* |
| *MYH11* | *MREG* |
| *NR4A1* | *MSH2* |
| *PHYHIP* | *MSH6* |
| *PLN* | *MTERF3* |
| *PNPLA2* | *MTF2* |
| *RILP* | *NEMP1* |
| *SGCA* | *POLR2D* |
| *SOD3* | *QSER1* |
| *SORBS1* | *RBM8A* |
| *SYNPO2* | *REST* |
| *TAGLN* | *RPAP3* |
| *TAMALIN* | *RPRD1A* |
| *TNS1* | *TASOR2* |
| - | *TIA1* |
| - | *TOPBP1* |
| - | *WAPL* |
| - | *XPO1* |
| - | *ZMYM4* |
| - | *ZNF107* |
| - | *ZNF146* |
| - | *ZNF260* |
| - | *ZNF512B* |
| - | *ZNF567* |

**Table S8: List 8 SOD3 Pearson correlated genes ρ≥|0.34| (n=100).** List 8^+^ is also available at <https://maayanlab.cloud/Enrichr/enrich?dataset=41096a40b61b6d7d3503ce0b2440f771>, list 8^-^ is available at <https://maayanlab.cloud/Enrichr/enrich?dataset=6ff34652a456cb44b0ca96bd688109ff>.

| **8^+^ (n=32)** | **8^-^ (n=68)** |
| --- | --- |
| *ADCY4* | *ABRAXAS2* |
| *AOC3* | *ACTR6* |
| *CDC42EP2* | *ADNP* |
| *CHRDL2* | *ADSS2* |
| *CLDN5* | *ANGEL2* |
| *CNN1* | *ATF7IP* |
| *DES* | *BRWD1* |
| *FOXF1* | *BTF3L4* |
| *GPBAR1* | *CAND1* |
| *HMCN2* | *CHTOP* |
| *HSPB6* | *CNOT6* |
| *HSPB7* | *COMMD2* |
| *IRAG1* | *CTDSPL2* |
| *KCNMB1* | *DHX9* |
| *LIMS2* | *DNAJC9* |
| *LMOD1* | *DUSP11* |
| *MUC3B* | *DYNLT2B* |
| *MYH11* | *FBXO28* |
| *NR4A1* | *FRMD8* |
| *PHYHIP* | *FXR1* |
| *PLN* | *G2E3* |
| *PNPLA2* | *HAUS3* |
| *PRELP* | *HLTF* |
| *RILP* | *HNRNPC* |
| *SGCA* | *IFT25* |
| *SOD3* | *KANSL2* |
| *SORBS1* | *KDM2A* |
| *SORBS3* | *MBD4* |
| *SYNPO2* | *MREG* |
| *TAGLN* | *MSH2* |
| *TAMALIN* | *MSH6* |
| *TNS1* | *MTERF3* |
| - | *MTF2* |
| - | *MYNN* |
| - | *NEMP1* |
| - | *NOL11* |
| - | *NUP153* |
| - | *PHIP* |
| - | *POLR2D* |
| - | *QSER1* |
| - | *RBM8A* |
| - | *REST* |
| - | *RFC4* |
| - | *RFC5* |
| - | *RPAP3* |
| - | *RPRD1A* |
| - | *S100PBP* |
| - | *SGPL1* |
| - | *SMC4* |
| - | *SRSF10* |
| - | *TASOR2* |
| - | *TIA1* |
| - | *TIMELESS* |
| - | *TMEM69* |
| - | *TOPBP1* |
| - | *TRA2B* |
| - | *USP37* |
| - | *USP46* |
| - | *VCPKMT* |
| - | *WAPL* |
| - | *XPO1* |
| - | *ZCCHC8* |
| - | *ZMYM4* |
| - | *ZNF107* |
| - | *ZNF146* |
| - | *ZNF260* |
| - | *ZNF512B* |
| - | *ZNF567* |
