# Supplementary material for: Elucidating the roles of SOD3 correlated genes and reactive oxygen species in rare human diseases using a bioinformatic-ontology approach
Source: PLoS One. 2024 Oct 31;19(10):e0313139. doi: 10.1371/journal.pone.0313139 (PMC11527182; doi:10.1371/journal.pone.0313139)
Supplement: S3 File — (DOCX) [file pone.0313139.s003.docx]

# Supplementary file 3: Experimental data show the novel gene CNN1 is dysregulated in rare disease Classical-like Ehlers-Danlos Syndrome type 1

Our study set out as a bioinformatic and ontology-based research to elucidate the potential roles of SOD3-correlated genes in human diseases. Through rigorous statistical testing and highly selective/restrictive filtering, we identified novel genes that may be involved in certain human rare diseases. This supplementary serves as an exemplar case, where we provide further experimental evidence to support the predictions made in the study. In particular, we prosed in the main paper that CNN1 might be a novel gene for Classical-like Ehlers-Danlos Syndrome type 1 (cEDS) and it should be further investigated as a potential biomarker.

First, we reasoned about what would be a valid experiment to support our prediction above. Ideally, cEDS patients should be recruited and compared with appropriate healthy controls, to investigate the differential expression of CNN1 between the disease and healthy states. However, the fact this is a rare disease means that the recruitment of cEDS patients in good numbers with sufficient statistical power will be a challenging task. Such a study involving patients and clinical samples is inherently resource-demanding, perhaps requiring a multi-centred research programme involving specialist hospitals or clinics for the rare disease.

As a group of Bioinformatics researchers, we don’t have the scope and resources to conduct clinical or lab experimental studies. Nevertheless, we focused on analysing publicly available experimental data for this disease to support our prediction. The GEO Series GSE264169 (https://www.ncbi.nlm.nih.gov/geo/query/acc.cgi?acc=GSE264169) contains RNA-Seq gene expression data on a mouse model of the cEDS. This mouse model of cEDS, created by conditional knockout of the Col5a1 gene, remarkably phenocopies wound healing defects in human cEDS. We obtained the raw RNA-seq counts data from this GEO Series, which were then processed using the TPM (Transcript Per Million) quantification method (https://pubmed.ncbi.nlm.nih.gov/34158060/), and finally transformed to log2(TPM+1) as the measure of gene level. Differential expression analyses between the cEDS disease (cKO mice) and wildtype (WT) control mice was thus performed on the log2 transformed gene expression data. Unlike typical RNA-seq differential expression analysis genome-wide, here we only focused on the particular genes of interest to validate our prediction, namely, Sod3, Cnn1, and Tgfbs, which are orthologous to the human genes SOD3, CNN1, and TGFBs, respectively.

Table S9 below shows the gene expression levels compared between the KO mice vs WT mice. Sod3, Cnn1, Tgfb1, Tgfb2, and Tgfb3 are all differentially expressed to the conventional level of statistical significance (p < 0.05 = alpha). As we focused on the 5 genes here, some form of multiple testing correction is needed to properly control false positives in this exercise of multiple hypothesis testing. It is clear from Table S9, that the differential expression for each of the 5 genes remains statistically significant (p < 0.01 = 0.05/5) after the p-value threshold has been corrected using the very conservative Bonferroni correction method. Under the Bonferroni method, the corrected p-value threshold = alpha / n, where alpha is conventionally level of statistical significance (0.05) and n is the number of hypotheses being tested.

Table S9: Differential gene expression between the cEDS disease condition (cKO mice) and healthy control (WT mice) for the five genes of interest.

| Gene | n_c | n_t | mean_c | mean_t | diff | CI_lwr | CI_upr | p.Ttest | p.Utest |
| --- | --- | --- | --- | --- | --- | --- | --- | --- | --- |
| Sod3 | 7 | 7 | 3.6 | 6.7 | 3.1 | 2.3 | 4.0 | 2.4E-05 | 0.00058 |
| Tgfb2 | 7 | 7 | 2.3 | 4.4 | 2.1 | 1.4 | 2.9 | 4.0E-05 | 0.00058 |
| Cnn1 | 7 | 7 | 1.7 | 5.5 | 3.8 | 2.3 | 5.4 | 0.00029 | 0.00058 |
| Tgfb3 | 7 | 7 | 3.5 | 4.9 | 1.4 | 0.7 | 2.1 | 0.0018 | 0.00117 |
| Tgfb1 | 7 | 7 | 4.7 | 6.4 | 1.8 | 0.8 | 2.8 | 0.0021 | 0.00058 |

Caption for Table S9 column headers: n_c is the number of wildtype mice in the control group; n_t is the number of cKO mice in the disease group; mean_c and mean_t are the mean expression levels for the two groups of mice, respectively; diff = mean_t – mean_c; CI_lwr and CI_upr are the lower and upper limits of the 95% confidence interval for diff; p.Ttest is the p value from Welch’s T test; p.Utest is the p value from the Mann-Whitney U test.

Figure S1 shows box plots of these genes’ expressions between the disease group and control group: (A) for Cnn1 gene; (B) for Sod3; (C) for Tgfb1; (D) for Tgfb2; and (E) for Tgfb3.

A


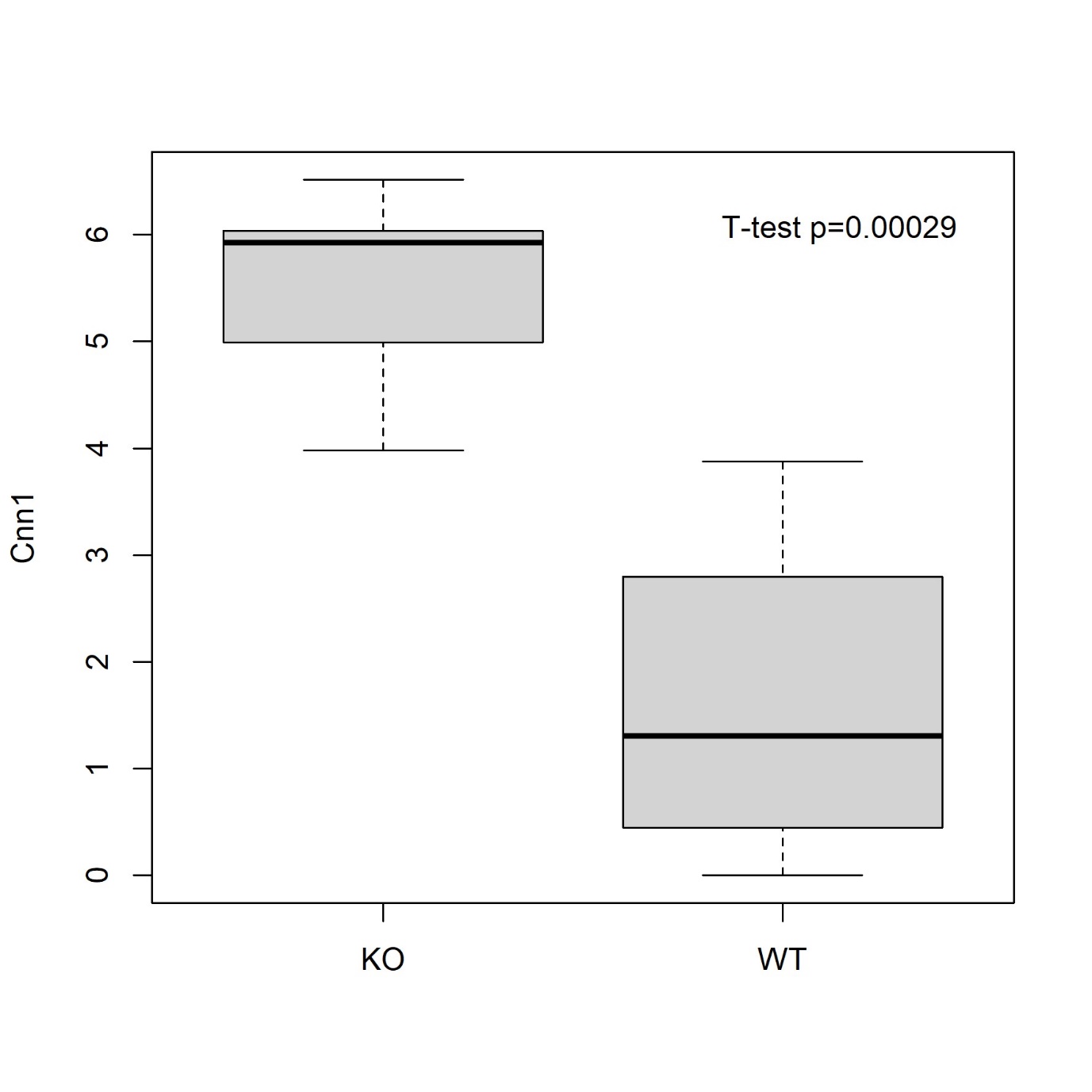


B
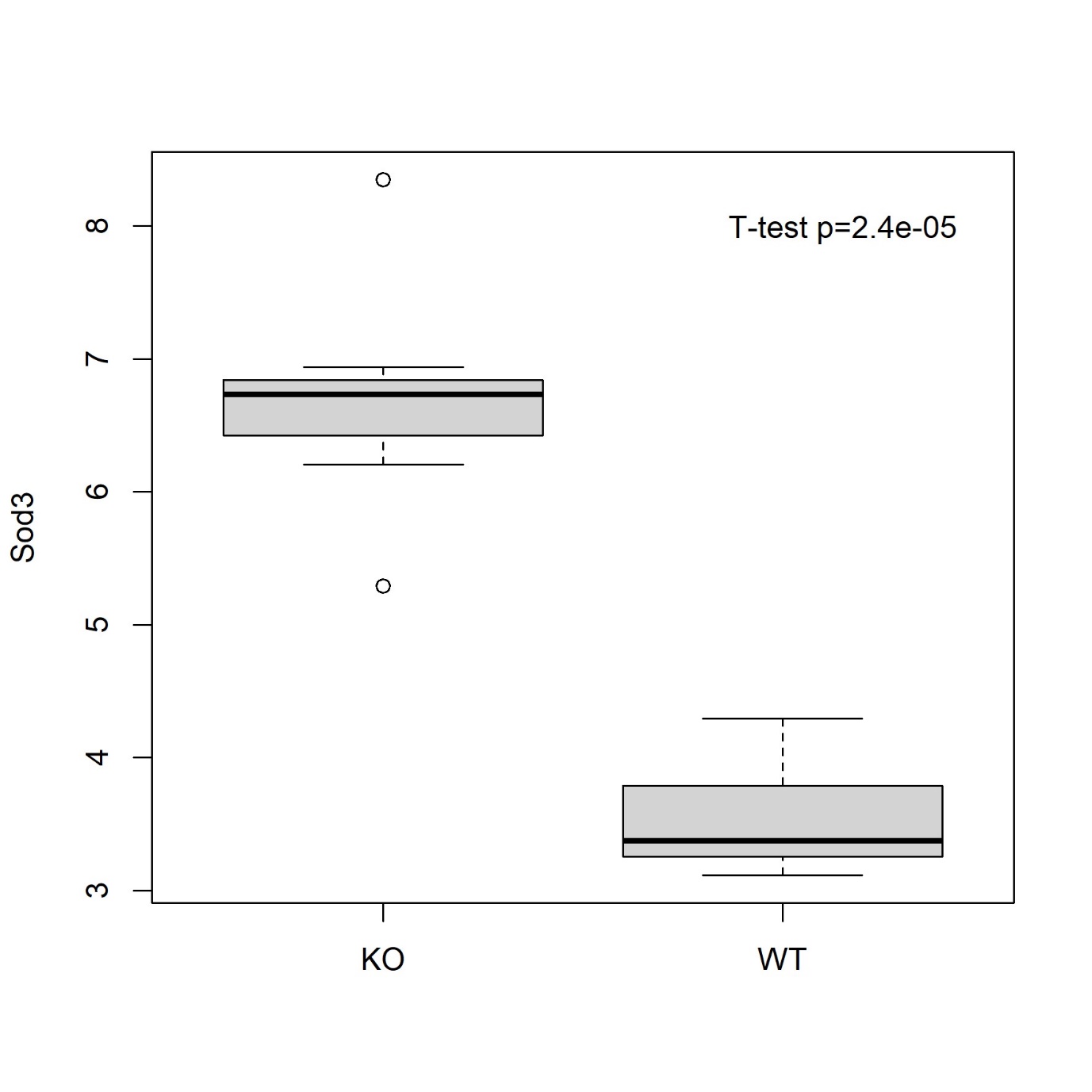


C


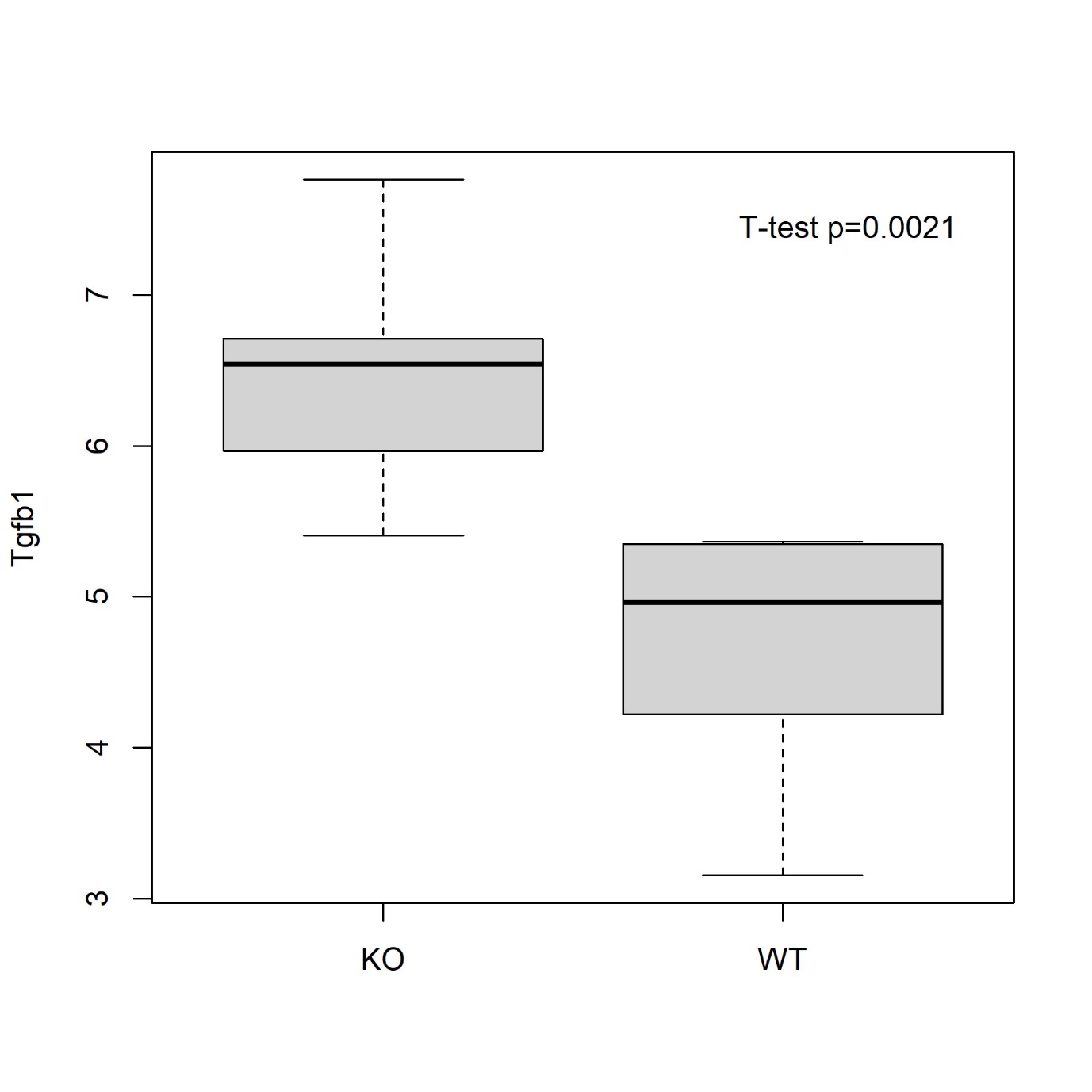


D


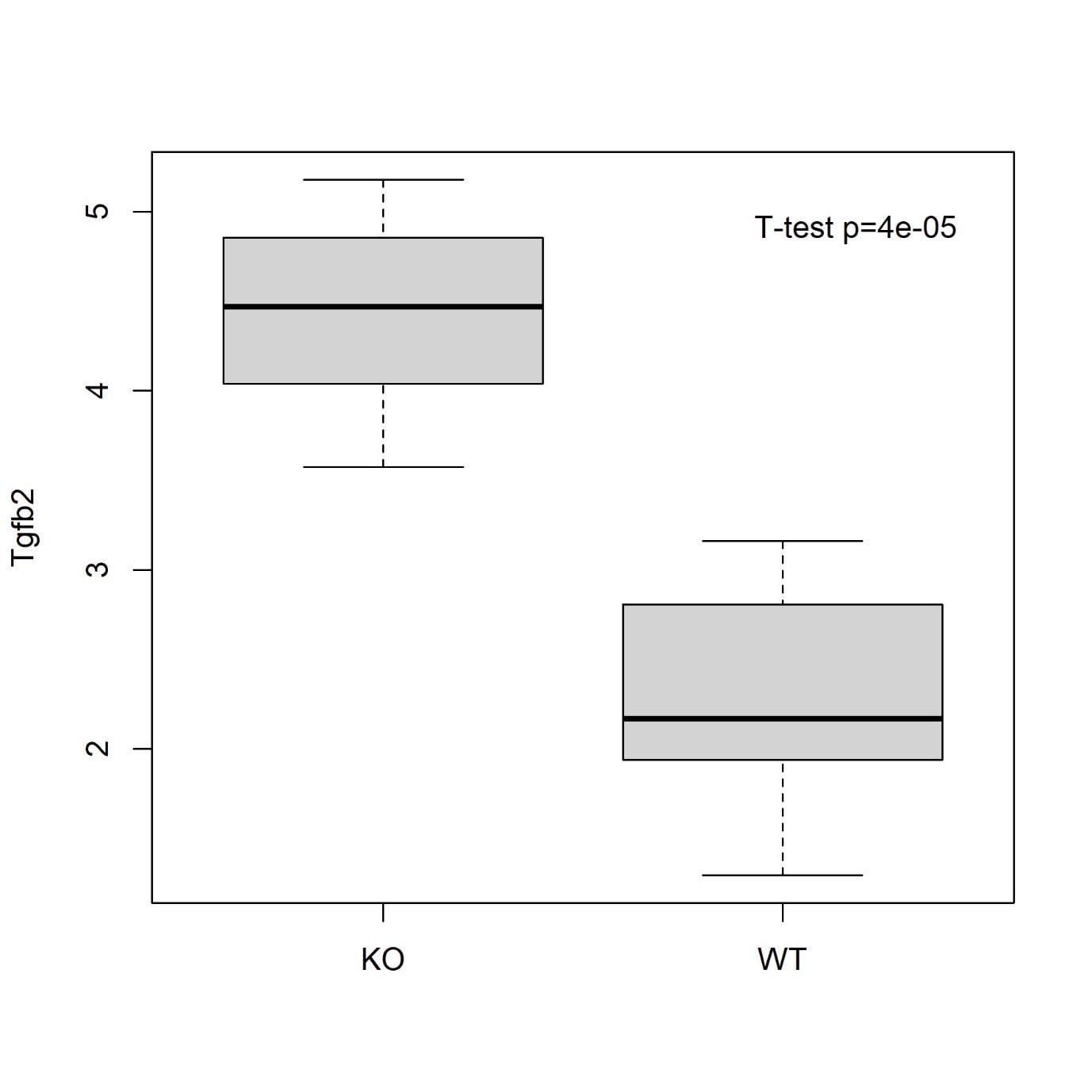


E


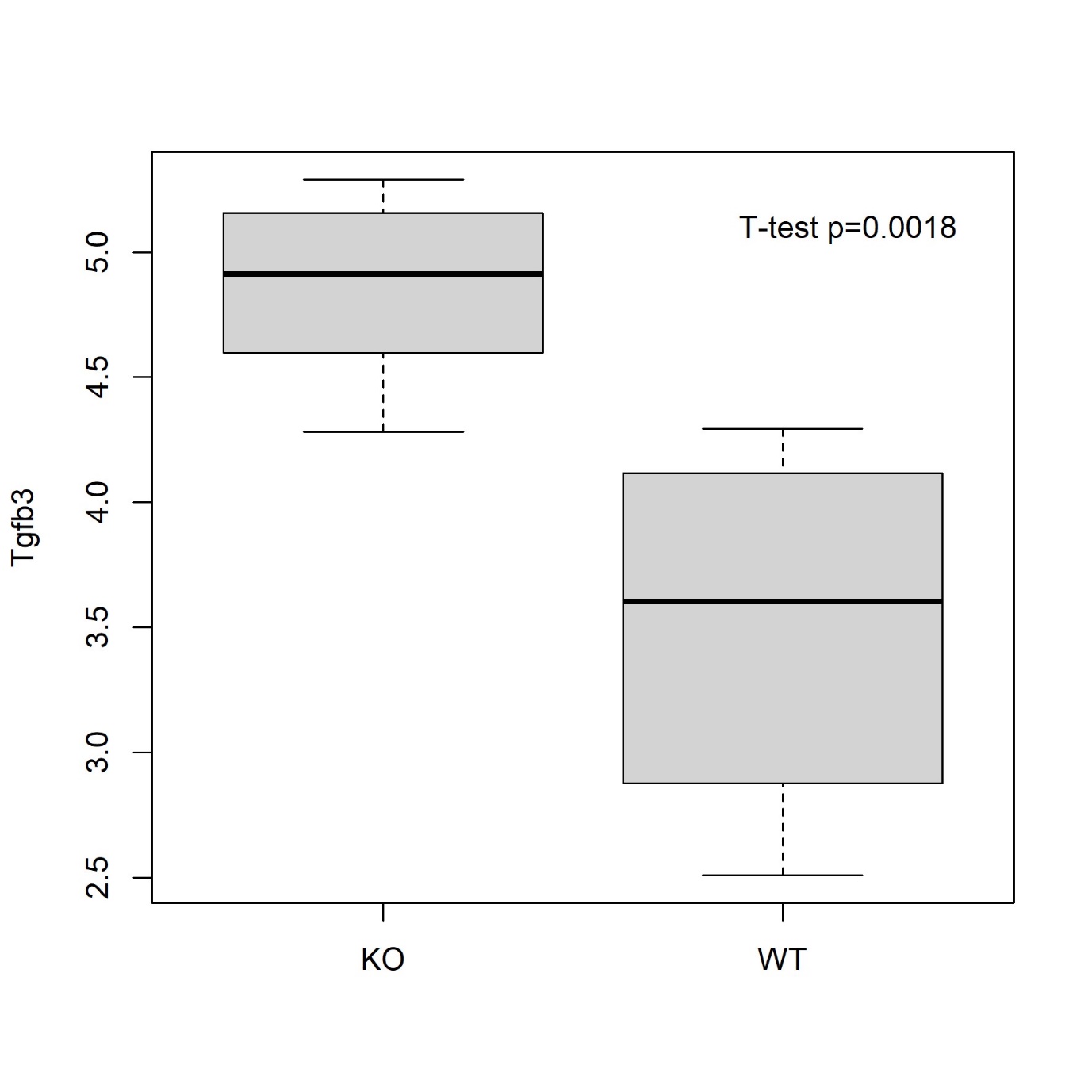


While our research had its specific remit as a bioinformatics and ontology-based study, we recognise the limitations of such a study in that that laboratory experimental validation is beyond the scope and capacity of our research group as bioinformatics researchers. Nevertheless, in this supplementary file, using publicly available experimental data, we have provided some experimental evidence to support the case that CNN1 is involved cEDS. This is novel because, up to now and to the best of our knowledge, no one else has suggested CNN1 as a gene related to cEDS, nor has anyone else reported this novel gene being dysregulated in this rare disease. Further investigations are needed to firmly establish the role of CNN1 in cEDS, which likely require large-scale clinical studies involving cEDS patients or more sophisticated laboratory experiments to gain deeper mechanistic insights.
